# How Viral and Intracellular Bacterial Pathogens Reprogram the Metabolism of Host Cells to Allow Their Intracellular Replication

**DOI:** 10.3389/fcimb.2019.00042

**Published:** 2019-03-04

**Authors:** Wolfgang Eisenreich, Thomas Rudel, Jürgen Heesemann, Werner Goebel

**Affiliations:** ^1^Chair of Biochemistry, Department of Chemistry, Technische Universität München, Garching, Germany; ^2^Chair of Microbiology, Biocenter, University of Würzburg, Würzburg, Germany; ^3^Max von Pettenkofer-Institute, Ludwig Maximilian University of Munich, Munich, Germany

**Keywords:** metabolic adaptation, viruses, intracellular bacterial pathogens, metabolism of infected and uninfected host cells, reprogamming of host cell metabolism

## Abstract

Viruses and intracellular bacterial pathogens (IBPs) have in common the need of suitable host cells for efficient replication and proliferation during infection. In human infections, the cell types which both groups of pathogens are using as hosts are indeed quite similar and include phagocytic immune cells, especially monocytes/macrophages (MOs/MPs) and dendritic cells (DCs), as well as nonprofessional phagocytes, like epithelial cells, fibroblasts and endothelial cells. These terminally differentiated cells are normally in a metabolically quiescent state when they are encountered by these pathogens during infection. This metabolic state of the host cells does not meet the extensive need for nutrients required for efficient intracellular replication of viruses and especially IBPs which, in contrast to the viral pathogens, have to perform their own specific intracellular metabolism to survive and efficiently replicate in their host cell niches. For this goal, viruses and IBPs have to reprogram the host cell metabolism in a pathogen-specific manner to increase the supply of nutrients, energy, and metabolites which have to be provided to the pathogen to allow its replication. In viral infections, this appears to be often achieved by the interaction of specific viral factors with central metabolic regulators, including oncogenes and tumor suppressors, or by the introduction of virus-specific oncogenes. Less is so far known on the mechanisms leading to metabolic reprogramming of the host cell by IBPs. However, the still scant data suggest that similar mechanisms may also determine the reprogramming of the host cell metabolism in IBP infections. In this review, we summarize and compare the present knowledge on this important, yet still poorly understood aspect of pathogenesis of human viral and especially IBP infections.

## Introduction

Viruses are obligate intracellular parasites and their reproduction entirely relies on the host cell machinery for the synthesis of viral components such as nucleic acids, proteins and membranes. Most viruses consist of single-stranded RNA or double-stranded DNA genome which is surrounded by either capsid proteins (non-enveloped viruses) or both capsid proteins and a lipid/protein membrane (enveloped viruses). After host cell attachment, viruses are internalized by clathrin-mediated endocytosis or micropinocytosis and subsequently escape from the endosomal vacuole into the cytosol. Here, the viral genome is released and transported to cellular compartments, where viral replication occurs: DNA viruses and some RNA viruses (e.g., influenza virus) enter the nucleus, whereas most RNA viruses remain in the cytosol (Mercer et al., 2010; Schelhaas, 2010; Heaton, 2017). After synthesis of the viral genome and proteins which assemble to new virus particles (virions), a complex release/egress process from the host cell is initiated: enveloped viruses egress by budding or exocytosis whereas most non-enveloped viruses are released by host cell lysis (Votteler and Sundquist, 2013; Weissenhorn et al., 2013; Khan et al., 2015). Virus formation depends on the metabolic capacity of the host cell to provide the necessary low molecular metabolites, i.e., nucleotides, amino acids and fatty acids (FAs)/lipids and energy in form of ATP. Recent studies (which will be discussed later) have shown that most viruses manipulate the host cell's metabolism in order to optimize the biosynthetic needs of the virus through “proviral metabolic changes.” The host cells, on the other hand, have developed metabolic strategies that inhibit viral replication through “antiviral metabolic changes” (Maynard et al., 2010; Yu et al., 2011; Netea et al., 2016).

In contrast, bacteria having their own macromolecular biosynthesis machinery rely—when replicating in host cells as in case of IBPs—to a considerable, albeit different extent on their metabolic capabilities for providing most low molecular catabolic as well as anabolic metabolites and ATP. Some of the IBPs are even metabolic generalists (e.g., *Salmonella* and *Shigella*), i.e., they may produce all low molecular metabolites by their own, starting from simple carbon-, nitrogen-, and sulfur sources and can efficiently grow in defined media containing only these basic nutrients. However, many IBPs have lost the genetic information for various catabolic and many anabolic pathways. The extremists among these latter IBPs, the so-called “obligate IBPs” (best known are the Chlamydiales and Rickettsiales), can efficiently proliferate only within suitable host cells. Yet, most human IBPs are “facultative IBPs,” i.e., they are able to grow extracellularly as well as intracellularly.

The ability to establish a stable intracellular bacterial live cycle depends on several bacterial and host cell functions: (I) Internalization by the host cell which requires (especially in case of non-professional phagocytic cells): (a) the recognition of matching host cell receptor(s) by IBP-specific surface components (“invasins”) and (b) after successful adhesion a triggered phagocytosis of the IBP. (II) Formation of specific pathogen-containing vacuoles (PCVs) or release of the IBP into the host cell's cytosol after lysis of the primary phagosome. (III) Avoidance of the host (cell) defense mechanisms. (IV) Reprogramming of the host cell's metabolism triggered by the IBPs. (V) Adaptation of the bacterial metabolism to that of the host cell. Whereas, significant progress has been made to unravel the processes (I) to (III) for most IBPs (Ribet and Cossart, 2015), much less is known concerning (IV) and (V).

The supply with suitable nutrients plays a crucial role for the intracellular survival and replication of IBPs. Considerable work has been therefore invested to answer the question which are the essential nutrients provided to the IBPs by host cells and how they are handled by the IBPs. Most of these investigations were carried out mainly in cell cultures and in part in animal models (Munoz-Elias and McKinney, 2006; Zimmermann et al., 2008; Eisenreich et al., 2010; Fuchs et al., 2012; Steeb et al., 2013; Abu Kwaik and Bumann, 2015; Bumann and Schothorst, 2017).

Interestingly, some of these studies show that facultative as well as obligate IBPs—so far analyzed with respect to intracellular nutrient consumption and metabolic fluxes—seem to follow within host cells a similar strategy which we termed “bipartite metabolism” (Grubmüller et al., 2014; Eisenreich et al., 2015). In short, “bipartite metabolism” means that the IBPs use as major energy source various host-derived energy-rich carbon compounds that are not as essential for the host cell as glucose. These include mainly C_3_-metabolites like pyruvate or glycerol, Ser, and Cys which can be converted to pyruvate (Eylert et al., 2008; Alkhuder et al., 2009; Grubmüller et al., 2014; Puckett et al., 2014; Abu Kwaik and Bumann, 2015; VanderVen et al., 2015; Häuslein et al., 2016, 2017; Chen et al., 2017; Mehlitz et al., 2017). Pyruvate is then further oxidized to acetyl-CoA, which feeds the tricarboxylic acid cycle (TCA) yielding important intermediates and ATP by oxidative phosphorylation (OXPHOS) or substrate phosphorylation (via acetyl-phosphate to acetate). It may also enter the gluconeogenesis pathway. Alternatively, FAs or cholesterol (CL) can be used as energy-rich components as in case of *Mycobacterium tuberculosis* (Mt). *De novo* biosynthesis performed by the IBPs within host cells is normally restricted to those compounds that cannot be provided by the host cells. This includes especially cell wall components. For the implementation of these indispensable biosynthetic pathways the IBPs seem to use limited amounts of host cell-derived glucose, glucose-6-phosphate, or other carbohydrates that can be converted to glucose-6-phosphate. Most other low molecular metabolites, including most amino acids, nucleotides, FAs and vitamins are mainly imported from the host cell. Exceptions are the three non-essential amino acids Ala, Asp, and Glu which are efficiently *de novo* synthesized by all IBPs tested (Eylert et al., 2008; Grubmüller et al., 2014; Häuslein et al., 2016, 2017; Chen et al., 2017; Mehlitz et al., 2017). It is interesting to note that these amino acids (in their D-forms) are either directly needed in considerable amounts for the synthesis of cell wall components (peptidoglycan, PG, and lipoteichoic acids) or act, like Asp, as precursor of meso-diaminopimelate (mDAP) which represents an essential building block of PG and is synthesized *de novo* by all IBPs except *Francisella*. The latter IBP probably uses Lys (which can be obtained from the host cell) instead of mDAP. Thus, the intracellular replication of the IBPs requires also a substantial amount of low molecular metabolites from the host cell. The “bipartite metabolism” strategy also allows the expression of the virulence factors that are essential for intracellular replication. Their expression is often under catabolite repression, i.e., blocked when glucose is the major carbon source (Eisenreich et al., 2013).

Compared to the considerable knowledge concerning the intracellular metabolism of IBPs, little is known about the reprogramming of the host cell metabolism necessary for efficient intracellular IBP replication (Eisenreich et al., 2017). In order to provide intracellular pathogens (viruses as well as IBPs) with the necessary amount of nutrients for a longer period of time, the host cell has to meet at least two important requirements: (a) essential nutrient transporters (especially for glucose and Gln) and major catabolic as well as anabolic pathways must be activated to meet the additional demand for nutrients by the IBPs; (b) premature cell death must be avoided in spite of the stress programs that might be triggered by the infection. Obviously, cancer cells and established cell lines (most of which derive from cancer cells) in general perform already an activated intermediary metabolism and thus fulfill these options, i.e., they show enhanced glucose and (often) Gln uptake, highly induced (aerobic) glycolysis, increased glucose flux through the pentose-phosphate pathway (PPP), and (often) enhanced anabolic activities which may lead to high-rate amino acid, nucleotide and FA/lipid biosynthesis (Ward and Thompson, 2012; Boroughs and DeBerardinis, 2015). It is therefore not surprising that these mammalian cell lines, often used as experimental host cells, allow efficient replication of many viruses and IBPs (Olivo, 1996; Eisenreich et al., 2013). However, these metabolic conditions normally do not apply for host cells which these pathogens encounter during *in vivo* infections. Most of these are terminally differentiated cells which are in a quiescent metabolic state, i.e., they show low-rate catabolic and anabolic activities. Other possible host cells may be in a metabolic activated state that is, however, adverse for the proliferation of most IBPs (e.g., classically activated M1-MPs, activated plasmacytoid dendritic cells, pDCs, and neutrophils). Exceptions are apparently lymphocytes, especially CD4^+^ T-cells and B-cells and alternatively activated M2-MPs; the activated metabolism of these immune cells allows efficient replication of some viruses (e.g., human immuno deficiency virus, HIV, in CD4^+^ T-cells and Epstein-Barr virus, EBV, in B-cells) and IBPs (e.g., *Salmonella, Brucella*, and other IBPs in M2-MPs) (Eisele et al., 2013; Xavier et al., 2013; Palmer et al., 2016).

However, in most virus and IBP infections, the metabolism of the encountered primary host cells must be first activated through the interaction of pathogen-specific factors with host cell targets to a “pro-microbial metabolic state,” to allow optimal progeny virion production and efficient IBP proliferation, respectively (Eisenreich et al., 2015, 2017; Goodwin et al., 2015; Sanchez and Lagunoff, 2015). While substantial information is meanwhile available on the viral and host factors leading to pro-viral metabolic host cell states, less is known on the corresponding interacting factors leading to pro-bacterial metabolic host cell states.

Since IBPs might pursue similar metabolic reprogramming strategies as viruses to reach replication-supporting metabolic states of the infected host cells, we will first present the known facts leading to pro- and anti-viral metabolic states, before we address the less clear corresponding conditions for IBPs. In this context, it is also intriguing to ask whether the frequently observed virus/IBP co-infections (McCullers, 2014; Kash and Taubenberger, 2015) might be favored by a pro-microbial metabolic background of host cells that is induced by one of the two intracellular pathogens.

## The Metabolism of Potential Mammalian Host Cells in Quiescent and Activated States

Mammalian cells, when cultured *ex vivo*, use glucose and Gln as major carbon and nitrogen sources, respectively, for cell proliferation. Their catabolism provides the cell (a) with the necessary precursors for the biosynthesis of nucleotides, non-essential amino acids and FAs/lipids required for the formation of DNA, RNA, proteins, and biomembranes, (b) with energy, and (c) with a balanced redox potential. The consumption of these two essential nutrients is tightly coordinated and regulated on several levels: (a) through specific nutrient sensing modules, (b) controlled transcription of genes encoding metabolic enzymes, (c) controlled translation of these transcripts, and (d) controlled posttranslational modifications of metabolic enzymes (Horton et al., 2002; DeBerardinis et al., 2008; Wellen et al., 2010; Thompson, 2011; Yin et al., 2012).

Starvation for glucose and Gln may lead to the consumption of alternative nutrients, like FAs and amino acids, especially of the branched chain amino acids, Ile, Val, and Leu (Boroughs and DeBerardinis, 2015). These nutrients can be provided to the cell by micropinocytosis and autophagy, processes that are stimulated by activated KRas and BRaf proteins (Commisso et al., 2013; Goldsmith et al., 2014). Their degradation may deliver acetyl-CoA by ß-oxidation of FA and succinyl-Co plus acetyl-CoA by degradation of branched-chain amino acids.

**Figure 1:**
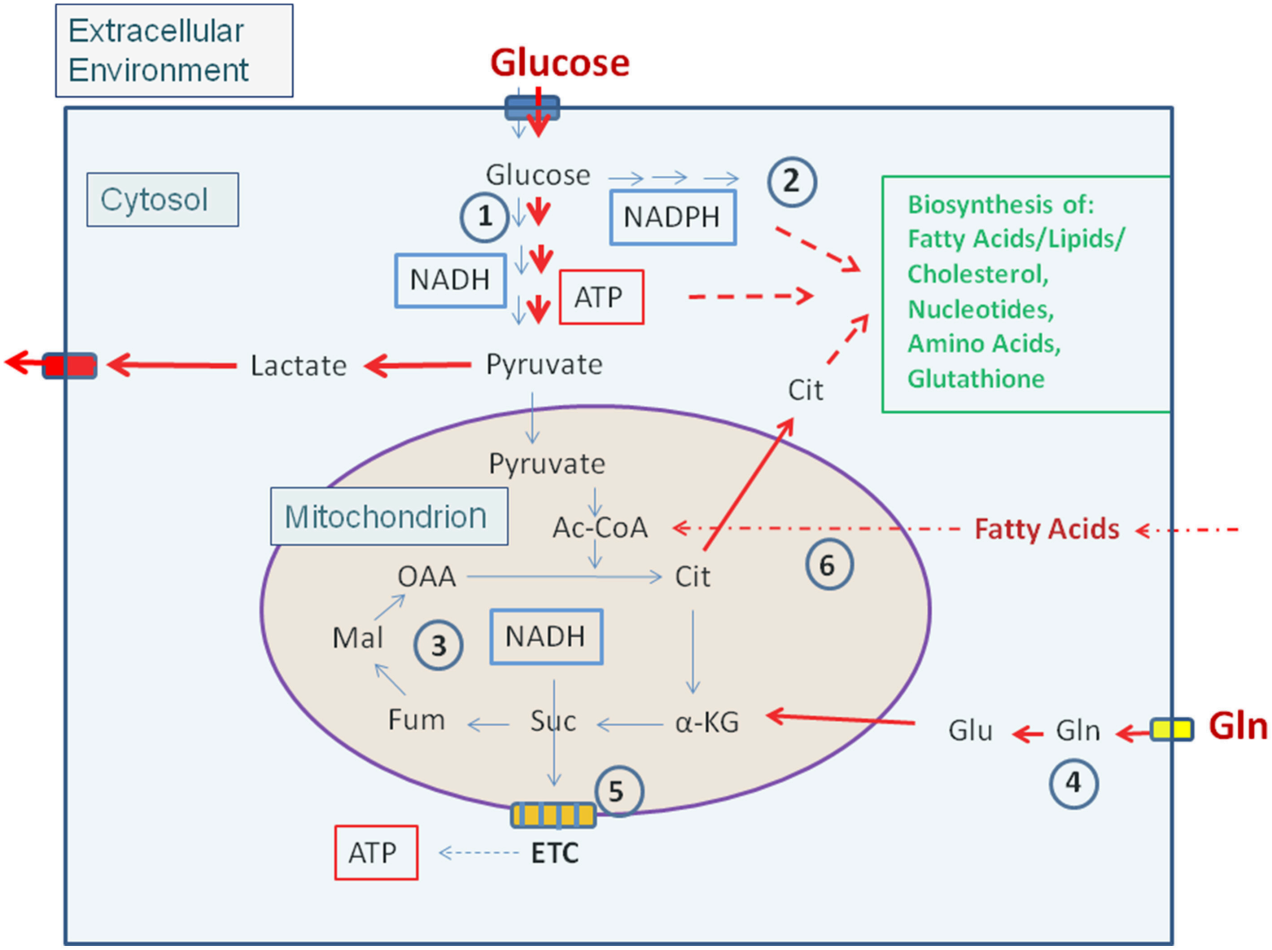
Carbon metabolism of mammalian cells in quiescent and activated states. In the quiescent state (thin blue arrows) a low amount of glucose, the major carbon source under these conditions, is taken up and oxidized mainly via the glycolytic pathway (1) and to a lesser extent by the (2). Pyruvate, the end product of glycolysis is transported to the mitochondria where it is further oxidized to CO_2_ through the TCA (3). NADH, NADPH, and FADH_2_, generated in (1), (2), and (3), respectively enter the electron transfer chain (ETC) where these electron donors are re-generated to NAD, NADP, and FAD thereby producing ATP by oxidative phosphorylation (OXPHOS) (5). ATP is also produced in the glycolytic pathway (1) by substrate phosphorylation. The anabolic pathways biosynthesizing the non-essential amino acids Ala, Ser, Asp, Asn, Glu, Gln, Pro as well as FAs, lipids, sterols, and nucleotides (green letters) are shut off or are running at a low level. In the activated state (red arrows), induced e.g., by growth factors, cytokines, activation of oncogenes, inactivation of tumor suppressors (see text for details), (1) and (2) are frequently highly induced, whereas (3) and (5) are now running at reduced levels. This metabolic condition is termed aerobic glycolysis or “Warburg effect.” In this state, pyruvate is converted to lactate thereby regenerating NAD which is needed for continuous glucose oxidation. Glutamine (Gln) and FAs may serve as alternative or additional carbon substrate(s) under these conditions. Gln is converted through glutaminolysis (4) to α-KG and FAs through ß-oxidation (6) to acetyl-CoA. Both metabolites can replenish the TCA. Under these conditions anabolic pathways are also activated as metabolites serving as precursors for the biosynthesis of amino acids, FAs/lipids/sterols, and nucleotides are produced in excess. (1): Glycolysis; (2): Pentose-phosphate pathway (PPP); (3): Tricarboxylic acid cycle (TCA); (4): Glutaminolysis; (5): Electron transfer chain/Oxidative phosphorylation (OXPHOS); (6): Fatty acid ß-oxidation (FAO). Ac-CoA, Acetyl-Coenzyme A; OAA, Oxaloacetate; Cit, Citrate; α-KG, α-ketoglutarate; Suc, Succinate; Fum, Fumarate; Mal, Malate. Blue box: Glucose transporters (GLUT-1-4), yellow box: glutamine transporter SLC1A5; ETC electron transfer chain, consisting of complexes I–IV and ATPase (complex V).

Most non-proliferating, terminally differentiated mammalian cells that may act as potential host cells for human viruses and IBPs (exclusively considered in this review) are, under low levels of growth factors, in a metabolically quiescent state. Under these conditions the cells adopt a mainly catabolic metabolism degrading glucose via the glycolytic pathway to pyruvate in the cytosol and oxidize most of it to CO_2_ in the mitochondrial TCA. The thereby formed NADH/H+ is channeled into the electron transfer chain (ETC) where oxygen is the final electron acceptor generating an electrochemical gradient which facilitates ATP production. Anabolic activities are very low under these conditions (Figure 1).

Enhanced growth factor concentrations, activation of (proto)oncogenes, or inhibition of tumor suppressors reprogram the cell metabolism toward a more pronounced anabolic mode which is crucial for cell growth and proliferation (Ward and Thompson, 2012). This metabolic program (Figure 1) includes increased nutrient uptake (mainly glucose often in combination with Gln) and increased flux of glucose through the glycolytic pathway and PPP, thereby generating essential building blocks for amino acid and nucleotide biosynthesis as well as NADPH necessary for driving reductive biosynthetic pathways (especially for nucleotides and FAs/lipids) and maintaining the redox homeostasis. In contrast to resting cells, activated cells produce ATP mainly by substrate phosphorylation and less by OXPHOS. Under these “aerobic glycolysis” conditions (also known as “Warburg effect”), most pyruvate, the end product of glycolysis, is converted to lactate which regenerates NAD essential for continued glucose oxidation. The flux of pyruvate into the mitochondrial TCA is often slowed down under these conditions, but enhanced glutaminolysis yields α-ketoglutarate (α-KG) which may supplement the TCA thus providing the precursors for the amino acids Asp, Asn, Glu, Gln, and Pro as well as citrate (Cit). Cit is transported into the cytosol where it is converted to oxaloacetate (OAA) and acetyl-CoA by the ATP-dependent citrate lyase (ACL). This acetyl-CoA is predominantly used for FA/lipid biosynthesis in the cytosol (Figure 1).

**Figure 2:**
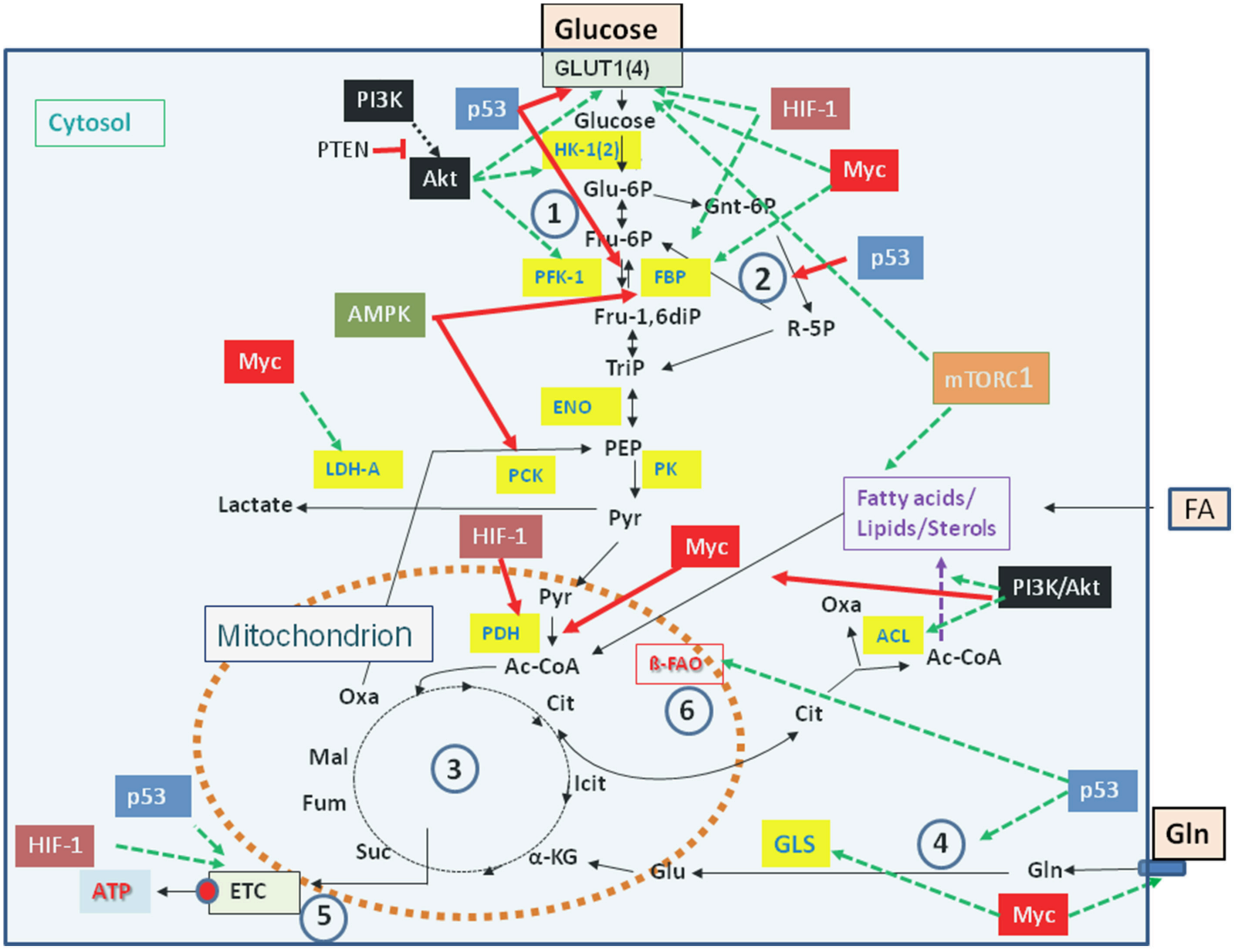
Major regulators controlling catabolic pathways by activating (green arrows) or inhibiting (red arrows) key enzymes (yellow boxes) and/or nutrient transporters. See text for details. Abbreviations of enzymes: HK-1(2), Hexokinase-1 and−2; PFK-1, Phosphofructokinase-1; FBP, fructose 1,6-bisphosphatase; ENO, phosphopyruvate hydratase (enolase); PK, pyruvate kinase; PDH, pyruvate dehydrogenase; LDH, lactate dehydrogenase A; ACL, ATP-dependent citrate lyase; GLS, glutaminase; ß-FAO, fatty acid ß-oxidation. For further abbreviations see Figure 1.

Carbon and energy metabolism is regulated by a complex net of nutrient sensors, growth hormone receptors, several downstream signaling pathways, and canonical transcription factors such as the nuclear factor “kappa-light-chain-enhancer” of activated B-cells (NF-kB), the activator protein 1 (AP-1), the nuclear factor of activated T-cells (NFAT), and others (Postic et al., 2007; Thompson, 2011; Ganeshan and Chawla, 2014; Pollizzi and Powell, 2014). Studies in the last decade especially on the dysregulated metabolism of cancer cells revealed the significance of oncogenes and tumor suppressor proteins as key regulators of cellular metabolism (Jones and Thompson, 2009; Courtnay et al., 2015; Camarda et al., 2017; Palm and Thompson, 2017). Important players are in particular the phosphoinositide-3-kinase (PI3K), the protein kinase B (Akt), the mammalian target of rapamycin (mTOR) complex 1 (mTORC1), the AMP-activated protein kinase (AMPK), the Ras proteins, the hypoxia-inducible transcription factor 1 (HIF-1), the myelocytomatosis oncogene (Myc), and the protein p53 (Figure 2). Whereas the impact of these factors and pathways on the regulation of growth cycle, differentiation, proliferation, immune responses, survival and apoptosis of mammalian cells were already extensively studied in the past, their involvement in the regulation of central metabolic pathways was recognized more recently (Yin et al., 2012; Iurlaro et al., 2014).

Mutations leading to permanently activated PI3K, Akt, KRas, or HIF-1α cause increased glucose uptake, enhanced glucose oxidation via aerobic glycolysis accompanied by increased lactate production and secretion, as well as enhanced PPP (Osthus et al., 2000; Semenza, 2010; Schodel et al., 2011; Vanhaesebroeck et al., 2012; Ying et al., 2012). In addition, Myc and KRas promote Gln uptake and glutaminolysis thereby supplementing the TCA (Wise et al., 2008; Son et al., 2013). This may enhance the efflux of Cit into the cytosol and its conversion to acetyl-CoA and OAA by ACL (Figure 2). Activation of these catabolic pathways favors the efficient production of intermediates, that are necessary for the formation of precursors for amino acids, nucleotides, and FAs needed for the generation of macromolecules (proteins, DNA, RNA) and biomembranes.

mTORC1 is a master regulator that drives anabolic cell growth. It senses amino acid levels and couples amino acid availability to growth or autophagy (Inoki et al., 2012; Jewell et al., 2013). Amino acids are important stimuli of mTORC1. The amino acid transporter SLC38A9 is a direct functional component of the amino acid sensing machinery that controls the activation of mTORC1 and thus the rate of protein synthesis and cell growth (Rebsamen et al., 2015).

Tumor suppressors, such as p53, the phosphatase and tensin homolog (PTEN), the retinoblastoma protein (RB), sirtuins (SIRT)-3 and−6, as well as the liver kinase B1/AMP-activated protein kinase (LKB1/AMPK), which act at various metabolic key nodes (Shackelford and Shaw, 2009; Puzio-Kuter, 2011; Houtkooper et al., 2012; Song et al., 2012; Nicolay and Dyson, 2013; Bieging et al., 2014), normally prevent excessive catabolic and/or anabolic metabolism. But inactivation of these tumor suppressors will activate catabolic pathways, including glycolysis and glutaminolysis (Figure 1).

AMPK, induced by an increased AMP/ATP ratio, triggers catabolic and inhibits anabolic processes, e.g., it stimulates (upon glucose shortage) the uptake of FA into mitochondria and their subsequent β-oxidation, whereas acetyl-CoA carboxylase activity is inhibited by activated AMPK thus blocking fatty acid synthesis (FAS) (Marcinko and Steinberg, 2014). Activated AMPK also reduces global protein synthesis by antagonizing the kinase activity of mTORC1.

These metabolic regulatory factors and pathways may interact with each other and functionally influence each other under specific nutritional and growth conditions (Inoki et al., 2012; Yin et al., 2012; Courtnay et al., 2015; Eisenreich et al., 2017).

Some key catabolic intermediates and (iso)enzymes have also significant regulatory functions for key metabolic processes (especially those involved in host defense): (i) The glycolytic enzyme hexokinase 1 (HK-1) interacts with the NLRP3 inflammasome, thereby activating caspase 1 which generates mature inflammatory interleukins IL-1ß and IL-8 from the corresponding pro-cytokines (Moon et al., 2015). (ii) The glycolytic glyceraldehyde-3-phosphate dehydrogenase (GAPDH) binds to the mRNA encoding interferon-γ (IFN-γ) and inhibits its synthesis. Upon induction of glycolysis, GAPDH is released from this complex and recruited to the glycolytic pathway, allowing IFN-γ mRNA translation (Chang et al., 2013). (iii) Pyruvate kinase isoenzyme 2 (PKM2), induced by enhanced glycolysis, slows down the glycolytic flux to pyruvate and leads to accumulation of glycolytic intermediates necessary for several biosynthetic pathways (Luo et al., 2011). A pro-inflammatory function of PKM2 in inflammatory MPs has also been reported (Shirai et al., 2016). (iv) The carbohydrate kinase-like protein (CARKL), possessing sedoheptulose kinase activity, acts as key regulator for PPP. Induction of this enzyme (e.g., in M2-MPs) limits the flux through PPP (Haschemi et al., 2012).

The TCA intermediates Cit and succinate (Suc) as well as itaconic acid (generated from Cit) may also regulate metabolic processes. These intermediates accumulate when the TCA is interrupted behind Cit or Suc due to reduced expression of isocitrate dehydrogenase and Suc dehydrogenase, respectively which occurs e.g., in M1 MPs and activated DCs (Tannahill et al., 2013; Jha et al., 2015). Suc inhibits prolyl hydroxylases leading to stabilization of HIF-1α with the above described metabolic consequences (Tannahill et al., 2013). Excess Cit is transported into the cytosol enhancing the production of FAs/lipids. Finally, itaconic acid has a direct antibacterial effect and thus links metabolism to immunity (Michelucci et al., 2013).

The described regulatory factors and pathways controlling the carbon and energy fluxes and the expression and/or activity of key metabolic enzymes represent possible targets for viral and bacterial effectors. These interactions manipulate the host cell metabolism in a promicrobial (increased survival, growth, and proliferation) or antimicrobial manner. Proven and suggested interactions of viral and IBP factors with these regulatory devices of the cellular metabolism will be discussed in the following.

## Proviral Metabolic Reprogramming Induced by Viral Infections

Viruses replicate in different cell types and under different physiological conditions of their host cells. In some cells a given virus performs an efficient lytic infection producing a big load of viral progeny, whereas in other cells it may carry out a long-lasting eventually lifelong persistence (asymptomatic latent infection) or a persistence with recurrent symptomatic infection (Goodrum et al., 2012). The metabolic needs under these conditions are expected to be significantly different (Delgado et al., 2012).

Robust lytic replication of both DNA and RNA viruses (typical for non-enveloped viruses) depends on high supply of nucleotides, amino acids, ATP, and eventually FAs/lipids required for efficient synthesis and modifications of viral nucleic acids, proteins and membranes as part of viral envelopes and cytoplasmic replication complexes (den Boon et al., 2010).

Increased uptake and catabolism of suitable carbon sources, especially glucose, but also Gln and FAs/lipids (often in combination), by the infected cells are necessary to cope with the high viral need for these metabolites during proliferative infections. Persistent infections obviously require less energy and metabolites.

**Figure 3:**
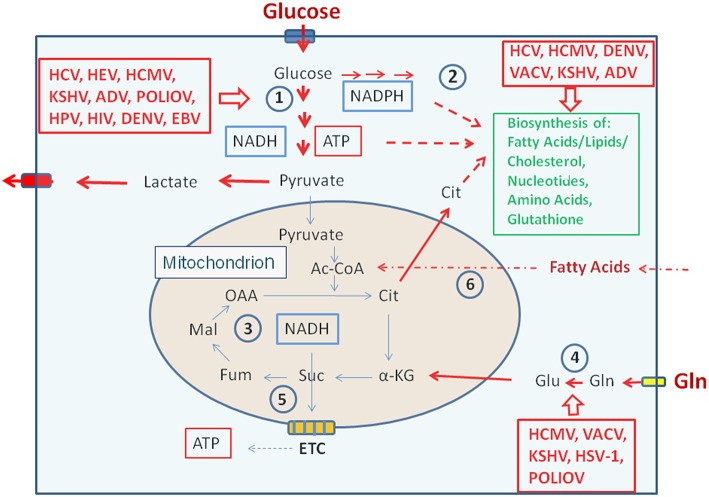
Metabolic pathways activated by viruses supporting their replication. **(Left box)** Viruses activating glucose uptake, glycolysis (1), PPP (2), and lactate production/secretion in their host cells; **(Right upper box)** Viruses activating biosynthesis of FAs/lipids or cholesterol and nucleotides, respectively in their host cells; **(Right lower box)** Viruses activating glutamine uptake and glutaminolysis (4). Abbreviations of viruses: ADV, Adenovirus; DENV, Dengue Virus; EBV, Ebstein-Barr Virus; HCMV, Human Cytomegalovirus; HCV, Hepatitis C Virus; HIV, Human Immunodeficiency Virus; HPV, Human Papillomavirus; HSV-1, Herpes Simplex Virus type 1; KSHV, Kaposi HSV-1, Herpes Simplex Herpesvirus; PolioV, Poliovirus; VACV, Vaccinia Virus. For further abbreviations see Figure 1 and text.

**Figure 4:**
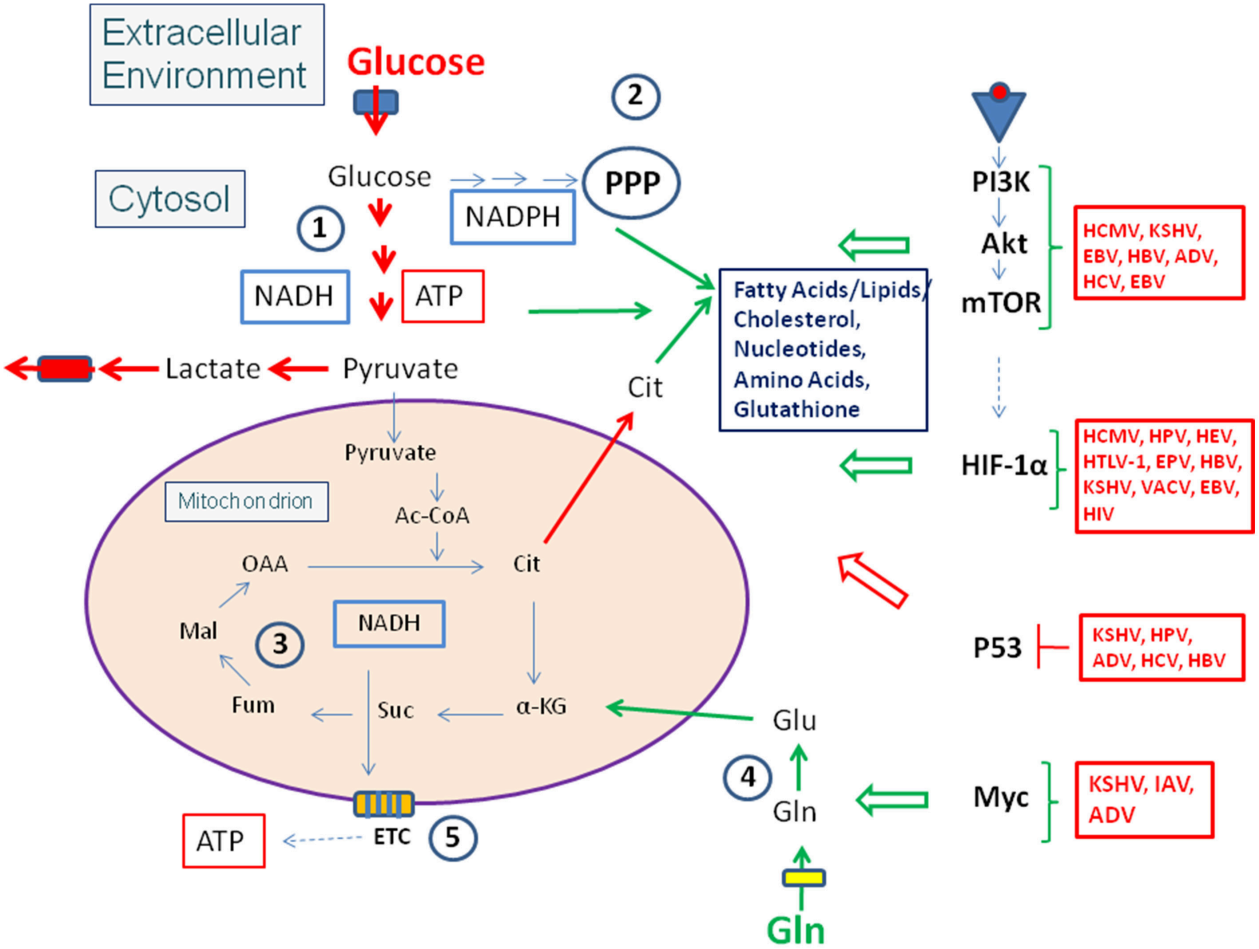
Viruses influence the activity of central metabolic regulators. Viral factors (see text and Tables 1, 2 for details) activate components of the PI3K/Akt/mTOR cascade or HIF-1α (see Figure 3) or inactivate the tumor suppressor p53. These interactions lead in general to enhanced glucose uptake, increased aerobic glycolysis and enhanced PPP activity as well as to activation of anabolic pathways in the infected host cells. Activation of Myc by some viral factors enhances especially Gln uptake and glutaminolysis. For abbreviations see Figures 1, 2.

Viruses pursue different strategies to meet these metabolic requirements, but most viruses interact at some point during their replication cycle with the PI3K/Akt/mTOR pathway (Figures 3, 4 and Tables 1, 2) through binding of viral factors to the p85 adaptor or the p110 catalytic subunit of PI3K in order to inhibit host cell death and/or to modulate cellular metabolism (Cooray, 2004; Buchkovich et al., 2008; Dunn and Connor, 2012; Diehl and Schaal, 2013). This signaling pathway is critically involved in the regulation of cell growth, (anti-)apoptosis, translation, but also the basic carbon metabolism (Courtnay et al., 2015) (Figure 2). Several other signal pathways and regulatory factors converge with the PI3K/Akt/mTOR pathway at various points thereby positively or negatively affecting these processes. Viral components may modulate directly or indirectly this pathway at different steps in a virus-specific manner as indicated in Figure 4 and Tables 1, 2.

**Table 1:** DNA-viruses: Interactions of viral factors and host cell targets that have been shown or are expected to cause metabolic reprogramming of the virus-infected cells.

**DNA viruses**	**Interacting viral components**	**Cellular targets**	**Used host cells and reference**
HCMV	IE1, IE2	Akt (+)	ts 13 cell line (mutant of the baby hamster kidney cell line BHK-21) (Yu and Alwine, 2002)
	Unknown	PI3K/Akt (+) HIF-1 (+)	Human foreskin fibroblasts (HFF2) and human fetal lung cells (HFL) (McFarlane et al., 2011)
	Unknown	PTEN (+)	Primary human aortic endothelial cells (HAEC) (Shen et al., 2006)
	pUL38	TSC/AMPK (+)	Human foreskin fibroblasts and 293T cell line (Moorman et al., 2008)
		mTORC1 (+)	(Brunton et al., 2013)
	pUL37x1	CaMKK/AMPK (+)	Primary human foreskin fibroblasts (Sharon-Friling et al., 2006)
	Unknown	Glycolysis, TCA, FAS (+)	MRC-5 fibroblast cell line and MDCK cell line (Munger et al., 2008)
	Unknown	AMPK (+)	MRC-5 fibroblast cell line (McArdle et al., 2012)
	Unknown	SREBP-1 (+)	Human foreskin fibroblasts (HFs) (Yu et al., 2012)
	Unknown	ChREBP (+)	Primary and life-extended human foreskin fibroblasts (Yu et al., 2014)
HSV-1	Unknown	Myc-induced GLS	Primary normal human bronchial epithelial cells (NHBE) (Thai et al., 2015)
	Unknown	Pyc (+)	Primary human foreskin fibroblasts (HFFs), ARPE19 human retinal pigment epithelial cell line, Vero green monkey kidney epithelial cell line, MRC-5 human embryonic lung fibroblast cell line (Vastag et al., 2011)
KSHV (HHV-8)	Unknown	HIF-1 (+)	Primary dermal human microvascular endothelial cells (HMVEC-d) and hTERT-TIME cell line (Delgado et al., 2010)
	LANA	p53 (–)	Renal carcinoma *VHL*-null cell line 786-O, human osteosarcoma *p53*-null cell line Saos-2, BJAB, DG75 and Louckes KSHV-negative type cell lines, and BC-3, BCBL-1, JSC-1 and BC-1 KSHV-positive cell lines (Cai et al., 2006)
	LANA	HIF-1 (+)	KSHV-positive cell lines (BCBL-1 and BC-3) and KSHV-negative type cells (BJAB and DG75), renal carcinoma *VHL*-null cell line 786-O, human osteosarcoma *p53*-null cell line Saos-2, and human embryonic kidney 293 (HEK293) and U2OS cell lines (Cai et al., 2007)
	miRNAs	EGLN2 and HSPA9 (–)	LEC, BCLB-1 cells latently infected with recombinant GFP KSHV, 293T, U2OS, and Vero cells (Yogev et al., 2014)
	Unknown	Neutral lipid synthesis (+)	HUVEC cells (Angius et al., 2015)
	Unknown	Myc induced glutaminolysis (+)	Tert-immortilized microvascular endothelial (TIME) cells and primary human dermal microvascular endothelial cells (hDMVECs) (Sanchez et al., 2015)
ADV	E1A and E1B	p53, RB (–)	Sf9 insect cell line and HeLa S3 cell line (Martin and Berk, 1998)
	E1A	Myc (+)	(Chakraborty and Tansey, 2009)
	E4-ORF1	PI3K (+)	Human epithelial cells (Kumar et al., 2014)
	E4-ORF1	Myc (+)	Epithelial cell line MCF10A and primary human bronchial epithelial (NHBE) cells (Thai et al., 2014, 2015)
EBV	LMP1	Glycolysis (+)	Immortalized NP69 nasopharyngeal epithelial cell line and other cell lines (Xiao et al., 2014)
		HIF-1 (+)	KH-1 and KH-2 cell lines (derived by fusion of HeLa and KR-4, and EBV-positive type III lymphoblastoid cell line) and HeLa cells (Kondo et al., 2006)
		HIF-1 (+)	MCF7 breast carcinoma cell line, B lymphoblastoid cell line (LCL), and peripheral B-cells (Darekar et al., 2012)
	BZLF1	p53 (–)	Human osteosarcoma cell line Saos-2, human epithelial 293T cells and 293/EBV cells, EBV-positive marmoset B lymphocytes B95-8 cells and Tet-BZLF1/B95-8 cells (Sato et al., 2009)
HPV-16 (18)	E6	p53 (–)	Primary human oral fibroblasts, primary human keratinocytes; NIH 3T3, Bosc23, Phoenix, and HaCaT cell lines (Tommasino et al., 2003)
		Akt/TORC1 (+)	Primary human foreskin keratinocytes (HFKs), human embryonic kidney cell lines HEK293 and 293T (HEK293 stably expressing the SV40 large T antigen), and human osteosarcoma cell line U2OS (Spangle and Munger, 2010)
		SGLT1 (+)	HeLa cells (Leiprecht et al., 2011)
		HIF-1 (+)	Epidermoid cervical carcinoma cell line (CaSki) and HEK293T cells (Guo et al., 2014)
	E6 and E7	HIF-1 (+)	Human lung cancer NSCLC cell lines, human adenocarcinomic epithelial cell line A549, human adenocarcinoma cell line and human lung cancer cell line H157 (Tang et al., 2007)
	E7	PKM2	Mouse embryo fibroblast cell line NIH 3T3 and 14/2 cell line (Zwerschke et al., 1999)
		PI3K/Akt (+)	NIH 3T3 cells, 14/2 cell line E7, and HEK293 cells (Pim et al., 2005)
		HIF-1 (+)	Neonatal foreskin keratinocytes (Bodily et al., 2011)
	E2	HIF-1 (+)	Human epithelial cell line C33-A and human osteoblast-like cell line Saos-2 (Lai et al., 2013)
SV40	T-Ag	p53 (–)	(Jiang et al., 1993)
			Fibroblast cell line CV-1 and primary AGMK cells (Yu et al., 2005)
		AMPK (+) and mTOR (+)	Human lymphatic endothelial cells (LEC), BCLB-1 cell line, U2Os cell line, and Vero cell line (Kumar and Rangarajan, 2009)
VACV	C16	HIF-1 (+)	Human immortalized microvascular endothelial (TIME) cells, primary dermal human microvascular endothelial cells (d-HMVECs), and primary human dermal BECs (Mazzon et al., 2015)
HBV	HBx	HIF-1 (+)	Chang X-34, HepG2, HeLa, HEK 293, and NIH3T3 cell lines (Yoo et al., 2008)
		Rab18(+)	Human HCC H7402, HepG2, and HEK 293T cell lines (You et al., 2013)
	Unknown	SREBP1/ACL (+)	HuH-7 and HepG2 cell lines (Teng et al., 2015)

*The host cells used in the studies are also indicated. ADV, adenovirus; EBV, Epstein-Barr virus; HBV, hepatitis B virus; HCMV, human cytomegalovirus; HPV, human papilloma virus; HSV-1, herpes simplex virus; KSHV (also called HHV-8), Kaposi's sarcoma-associated herpesvirus; SV40, simian virus 40; VACV, vaccinia virus. For abbreviations of the cellular targets see text; unknown means: the viral factor responsible for host cell target binding and activation has not yet been determined*.

**Table 2:** RNA-viruses: Interactions of viral factors and host cell targets that that have been shown or are expected to cause metabolic reprogramming of the virus-infected cells.

**RNA viruses**	**Interacting viral components**	**Cellular targets**	**Used host cells and references**
IAV	Unknown	Glycolysis (+)	MDCK epithelial cell line (Ritter et al., 2010)
		Myc (GLS) (+)	Primary human bronchial epithelial cells (NHBE)
	Unknown		(Thai et al., 2015)
		Myc (+)	(Smallwood et al., 2017)
		PI3K/Akt (+)	
		mTOR (+)	
RSV	Unknown	HIF-1 (+)	Primary human bronchial epithelial cells (HBEpC) (Kilani et al., 2004)
HCV	Unknown	AMPK (–)	Huh-7 cells (Mankouri et al., 2010)
		mTOR (+)	Immortalized human hepatocytes (IHH) and Huh7.5 cell line (Bose et al., 2012)
		HIF-1 (+)	UHCVcon-57.3, UCp7con-9.10, and UNS3-5Bcon-27 cell lines (Ripoli et al., 2010)
	NS5	HK-2 (+)	Huh-7.5 cell line (Diamond et al., 2010)
			Huh7.5, HepG2, and HEK 293T cell lines (Ramiere et al., 2014)
	NS3, NS5	RB and p53 (–)	(Lemon and McGivern, 2012)
			Huh-7.5 cell line
		PDK (+)	(Jung et al., 2016)
HEV	ORF3	PI3K/Akt (+) HIF-1 (+)	Huh7, ORF3/4nd pCN cell lines (Moin et al., 2009)
DENV	NS4A	Autophagy Lipid metabolism (+)	MDCK cells (McLean et al., 2011)
			Huh-7.5, Huh-7, HepG2 cell lines, and baby hamster kidney cell line BHK-21 (Heaton and Randall, 2010)
	NS3A	FASN (+)	Huh-7.5, HEL, HEK 293T, BHK-21, and Vero cells (Heaton et al., 2010)
	Unknown	Glycolysis (+)	Primary HFFs and TIME cells (Fontaine et al., 2015)
	NS1	GAPDH (+)	BHK-21 cells and human umbilical vein endothelial cells (HUVEC-C) (Allonso et al., 2015)
HIV-1	Unknown	GLUT-1 (+)	Human CD4+ T-cells (Palmer et al., 2016)
	Vpr	HIF-1 (+)	U937 MP cell line (Deshmane et al., 2009; Barrero et al., 2013)
	Vpr	Dysregulation of glutamate metabolism	U937 (Datta et al., 2016)
		Increase in glucose uptake and glycolysis in CD4 T cells, but decrease in U937 MPs	CD4 T-cells and U937 MP cell line (Hollenbaugh et al., 2011)
	Env	mTOR (+)	Human MOs, CD4+ T lymphocytes, myeloid DCs (MyDCs), Hut-CCR5, HEK 293T, and HeLa P4-R5 cell lines (Blanchet et al., 2010)
HTLV	Tax	PI3K/Akt (+)	T-cell leukemia cell lines MOLT-4 and CCRF-CEM, HTLV-1-infectd T-cell lines MT-2, MT-4, SLB-1 and HUT-102 (Tomita et al., 2007)

*The host cells used in the studies are also indicated. DENV, dengue virus; HCV, HEV, hepatitis C and E viruses; HIV, human immunodeficiency virus; HTLV, human T-lymphotropic virus; IAV, influenza A virus; RSV, respiratory syncytial virus*.

Another frequent target for viral factors affecting the host cell metabolism is AMPK. As pointed out above, activated AMPK stimulates energy-producing processes but inhibits energy-consuming anabolic processes, especially protein synthesis, by antagonizing mTOR kinase (Inoki et al., 2012). AMPK and mTOR are therefore crucial regulators for cellular metabolism, energy homeostasis and growth (Figure 2).

Viruses change the central carbon metabolism of the infected host cells (Figure 3), sometimes in a similar way as observed in many tumor cells (Figures 2, 4), i.e., by the permanent activation of cellular (proto)-oncogenes (e.g., Myc), the inactivation of tumor suppressors (e.g., p53) or by the introduction of virus-specific oncogenes as in case of certain tumor DNA viruses (e.g., large and small T antigens of simian virus 40, SV40) (Tables 1, 2) (Heaton and Randall, 2011b; Goodwin et al., 2015; Sanchez and Lagunoff, 2015; Levy and Bartosch, 2016; Mushtaq et al., 2016).

**Figure 5:**
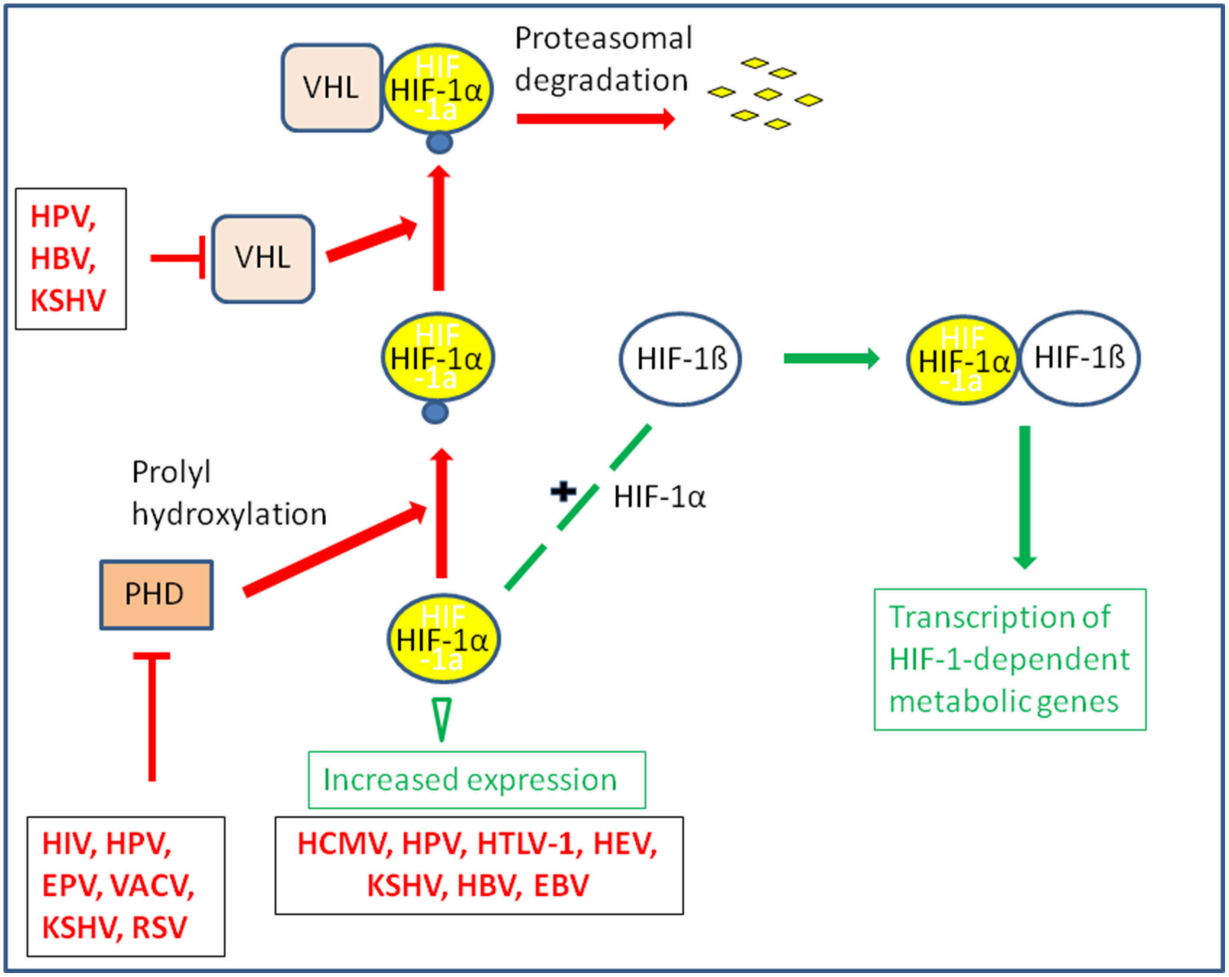
Some viruses activate HIF-1 by stabilization or increased expression of HIF-1α. The transcription factor HIF-1 is a heterodimer consisting of HIF-1α and the constitutively expressed HIF-1ß. Under normoxic conditions HIF-1α is hydroxylated by prolylhydroxylase (PHD) at conserved proline residues making HIF-1α recognizable for the Von Hippel-Lindau E3 ubiquitin ligase (VHL) complex which leads to rapid degradation by the proteasome. Some viruses are able to inhibit proteasomal degradation of HIF-1α even under normoxic conditions by inhibiting PHD or blocking association of HIF-1α with VHL; others may enhance expression of HIF-α.

The oxygen tension may also significantly affect the replication of several DNA and RNA viruses by modulating the rate of the host energy metabolism. This occurs often through stabilization of HIF-1α and manipulation of the HIF-1 pathway which, as further outlined below, represents also a frequent target for specific viral products (Vassilaki and Frakolaki, 2017) (Figures 4, 5 and Tables 1, 2).

Autophagy is in general an essential mechanism of host immune defense against viral infections by delivering viral antigens to the endosomal/lysosomal compartments for major histocompatibility complex (MHC)-mediated presentation or through direct elimination of the viruses by xenophagic degradation. But some viruses may actively subvert autophagy for their benefit by a variety of mechanisms, among others by providing additional nutrients for the host cell metabolism thereby supporting viral replication (Heaton and Randall, 2011a; Jordan and Randall, 2012; Dong and Levine, 2013). A well-studied example is the degradation of lipid droplets (LD) by induced autophagy and the use of the released triglycerides as carbon source for ATP production via ß-oxidation during Dengue virus (DENV) infection (Heaton and Randall, 2010).

These metabolic alterations, which may be caused by the interaction of virus-specific factors with these host cell targets, lead (depending on the virus, see below) to: (a) the induction of core catabolic pathways, i.e., glycolysis, PPP, TCA, ß-oxidation of FAs (FAO), as well as anabolic pathways, i.e., enhanced biosynthesis of nucleotides, FAs/lipids and amino acids; (b) induction of virus-specific biosynthetic processes (e.g., synthesis of virus-specific FAs and lipids) and modifications of viral components (e.g., virus-specific protein glycosylation or modifications of cellular FAs).

Together, the virus-mediated metabolic reprogramming force the host cells to provide increased amounts of nucleotides necessary for viral nucleic acid replication, of amino acids necessary for virion assembly and of FA/lipids required for membrane formation necessary for the viral replication machinery and eventually for membrane envelopes (in case of enveloped viruses). The increased generation of ATP is necessary for nucleic acid replication and virion packaging. In addition, virus-specific modifications of proteins (e.g., by glycosylation), nucleic acids and FAs may be required for the generation of infectious virus particles.

### Induction of Uptake and Catabolism of Glucose, Glutamine, and Fatty Acids by Interactions of Specific Viral Factors With Host Metabolic Targets

#### Glucose as Major Carbon Source

Most of the efficiently replicating DNA and RNA viruses activate the host cell metabolism by increasing glucose uptake and catabolism via glycolysis and PPP, generating ATP mainly by substrate phosphorylation and metabolites that support those host anabolic pathways (especially for amino acids, nucleotides, and FAs/lipids) necessary for the biosynthesis of viral nucleic acids, proteins, and FAs/lipids. In addition, the increased amount of NADPH_2_ generated by the oxidative arm of PPP suppresses oxidative stress by regeneration of glutathione, further emphasizing the importance of glucose for the proliferation of most viruses (Figure 3).

The mechanisms for achieving this goal include: (a) activation of central signaling cascades modulating cellular metabolism (especially of PI3K/Akt/mTORC1, HIF-1α, and AMPK; see Tables 1, 2) by specific viral factors (Buchkovich et al., 2008; Brunton et al., 2013), (b) inhibition or degradation of the tumor suppressor p53 by the interaction of specific viral proteins (Vprs) with p53 directly (thereby inhibiting the transcriptional activity of p53) or with the proteasomal degradation machinery (thereby enhancing degradation of p53) (Lazo and Santos, 2011; Zaika et al., 2015; Aloni-Grinstein et al., 2018), (c) direct interaction of viral factors with specific metabolic enzymes (Rabinowitz et al., 2011; Spencer et al., 2011; Allonso et al., 2015), and (d) interaction of viral factors with specific metabolic regulators, like the carbohydrate-responsive element-binding protein (ChREBP) and/or the sterol regulatory element-binding protein (SREBP) (Yu et al., 2012).

These interactions activate directly or indirectly glucose uptake, glycolysis, and PPP, (often) reduce OXPHOS, but also enhance anabolic pathways (Figure 4), especially lipogenesis and biosynthesis of nucleotides, as exemplified in the following for some viruses:

Human cytomegalovirus (HCMV), a DNA virus, actively replicates in fibroblasts and epithelial cells where it induces uptake and catabolism of glucose but also increased import of Gln and enhanced glutaminolysis accompanied by efficient FA/lipid synthesis (Vastag et al., 2011). In primary hematopoietic progenitor cells (e.g., CD14^+^ MOs) carrying out low FAS (Ecker et al., 2010), HCMV maintains latency, but upon differentiation of the MOs into MPs which show induced FAS, HCMV is reactivated to produce infectious progeny (Goodrum et al., 2002; Cheung et al., 2006). Thus, not only glucose consumption but also the level of FAS in the host cell appears be a decisive factor for the replication state of HCMV within infected cells.

However, even closely-related viruses may activate the metabolism in different ways: the HCMV-related herpes simplex virus 1 (HSV-1) does not activate glucose uptake and glycolytic flux. Furthermore, whereas HCMV induces the influx of acetyl-CoA into the TCA thus promoting FAS, HSV-1 stimulates the influx of pyruvate into the TCA through pyruvate carboxylase (Pyc) generating OAA and thereby feeds the pyrimidine biosynthesis (Vastag et al., 2011).

The Kaposi's sarcoma (KS)-associated herpes virus (KSHV), the etiologic agent of KS, establishes a predominantly latent infection in the main KS tumor cell type, the spindle cell, which is of endothelial cell origin. KSHV requires the induction of multiple metabolic pathways (Tables 1, 2), including glycolysis (Carroll et al., 2006; Delgado et al., 2010) and FAS (Angius et al., 2015) for the survival of latently infected endothelial cells.

EBV, an oncogenic human herpes virus, productively infects B cells and epithelial cells and persists in a latent state in memory B-cells of the peripheral blood (Price and Luftig, 2014). EBV has been linked to the development of B-cell and epithelial malignancies. Early infection, accompanied by hyperproliferation of the virus, is characterized by increased glucose import (via induction of the glucose transporter-1, GLUT-1), and glycolysis, but also by active OXPHOS and suppression of autophagy. In the arrested cells, a decreased expression of genes involved in the TCA and OXPHOS is observed. These cells show increased expression of p53 regulated genes, which causes activation of AMPK, reduction of mTOR signaling, and, consequently, elevated autophagy which appears to be important for host cell survival (McFadden et al., 2016).

Enveloped viruses like hepatitis C virus (HCV) and influenza A virus (IAV), as well as non-enveloped viruses like poliovirus, coxsackievirus, and rotavirus depend on increased glucose uptake and FAS for efficient viral proliferation (Gaunt et al., 2013; Sanchez and Lagunoff, 2015; Xie et al., 2015). For these viruses, the increased *de novo* FAS is not only necessary for the viral envelope, but is also required (in addition to the recruitment of cellular membranes) for the formation of the cytosolic virus-specific replication complexes (den Boon et al., 2010).

In some DNA tumor viruses (e.g., adenoviruses, papilloma viruses), one specific viral factor (e.g., E4-ORF of adenovirus, ADV, or E6 and E7 of human papilloma virus, HPV-16) may interact with more than one host cell target controlling glucose metabolism. In others, different proteins of the same virus can interact with different cell targets, all of which may enhance glucose uptake and glycolysis, like glucose transporters, HIF-1, and Myc (Mushtaq et al., 2016) (Figures 4, 5 and Tables 1, 2).

HIV-1 infects activated CD4^+^ T-cells, MOs and MPs. Increased glucose uptake (through GLUT-1 induction) and glycolysis are hallmarks of HIV infection in CD4^+^ T-cells and MOs (Hollenbaugh et al., 2011; Macintyre et al., 2014; Palmer et al., 2016). Metabolic reprogramming in HIV-1 infected MPs is strongly influenced by the Vpr which appears to induce HIF-1α (Datta et al., 2016). MPs treated with Vpr protein or infected with HIV-1 show increased glucose uptake and enhanced expression of critical glycolytic and TCA enzymes in response to Vpr in a HIF-1α dependent manner (Datta et al., 2016). This HIV-1 induced MP metabolism is accompanied by extracellular accumulation of α-KG and Gln (see also Tables 1, 2) (Barrero et al., 2013; Datta et al., 2016). The Vpr expression in HIV-1 infected MPs may also play an important role in the neuropathogenesis of the acquired immunodeficiency syndrome (James et al., 2016).

#### Glutamine as Major or Additional Carbon Source

Besides activating expression of glycolytic enzymes, Myc is also known to enhance Gln catabolism by increasing the expression of the Gln transporters ASCT2/SLC1A5 and SNAT5/SN2 and of glutaminase (GLS) (Wise et al., 2008; Gao et al., 2009). E4ORF1 protein of ADV activates Myc thereby inducing Gln catabolism which supports virus replication (Thai et al., 2015). Consequently, inhibition of GLS decreases replication of ADV in primary epithelial cells. GLS inhibition in these host cells also inhibits replication of HSV-1 and IAV, suggesting a similar dependence on Gln for the replication of these latter viruses. The molecular mechanism of how this occurs is, however still unknown for both viruses (Thai et al., 2015).

Vaccinia virus also relies mainly on induced Gln import and glutaminolysis of the host cell for its replication but does not induce glucose uptake and glycolysis (Fontaine et al., 2014).

HCMV activates, in addition to enhanced glycolysis (see above), also the uptake of Gln and glutaminolysis to replenish the TCA (Chambers et al., 2010). This is necessary, since the intermediates generated by glucose oxidation are mainly used for FA and nucleotide biosynthesis.

KSHV requires in the typical latent infection of spindle cells enhanced glycolysis and FAS, but enhanced Gln uptake and increased levels of intracellular Gln are also hallmarks of latent KSHV infection (Sanchez et al., 2015). For this latter goal, KSHV apparently activates Myc which up-regulates the Gln transporter SLC1A5 and glutaminolysis.

#### Degradation and/or Synthesis of Fatty Acids as Essential Pathways for Viral Replication

Mitochondrial FAO generates acetyl-CoA which, when introduced into the TCA, leads to generation of NADH/H+ and FADH_2_ necessary for maintaining OXPHOS. This catabolic pathway is required for the replication cycle of some viruses, including HCV (Diamond et al., 2010; Rasmussen et al., 2011), measles virus (MV), vesicular stomatitis virus (VSV), and Semliki Forest virus (Takahashi et al., 2007).

DENV relies on both, FAO and FAS, for active replication (Heaton et al., 2010). Two different non-structural DENV proteins, NS4A and NS3, respectively, are involved in the induction of autophagy of LD (yielding triglycerides) and the activation of the FA synthase (FASN), respectively (Heaton et al., 2010; McLean et al., 2011) (see also Tables 1, 2). While the triglycerides are used for energy production via FAO, FAS is apparently required for the formation of the DENV-specific cytosolic replication complex (in addition to the recruitment and remodeling of cellular membranes).

In addition to DENV, several other viruses induce and require FAS for efficient replication, including HCV (Kapadia and Chisari, 2005), HCMV (Munger et al., 2006), KSHV (Bhatt et al., 2012), and vaccinia virus (Greseth and Traktman, 2014).

Together, the virus-induced reprogramming of the host cells' metabolism in general supports viral replication. Mechanistically, the virus-induced metabolic programs are triggered by the interactions of one or more specific viral factor(s) with key regulatory factors and pathways as well as key metabolic enzymes that control the central carbon metabolism of the respective host cells (Figures 3, 4 as well as Tables 1, 2). These metabolic interventions seem to be virus- and host cell-specific and hence have to be clarified for each individual virus and each host cell type to identify crucial metabolic targets that may be of therapeutic benefit. However, it should also be mentioned that discrepancies in the metabolic reprogramming profiles of host cells upon viral infection have been reported in some instances. Such discrepancies could be caused by the different host cell types used in these studies (Palmer et al., 2016), especially when established cell lines are applied as host cells for viral infections which is indeed the case in many studies (see Tables 1, 2). These host cells are (in most cases) already metabolically (differently) activated which may obscure the modifications of cell metabolism occurring during viral infections *in vivo*. Therefore, we provide in Tables 1, 2 also the information which host cells are used in the studies cited.

## Modulation of the Cell Metabolism by Interferons (IFN) Leading to Antiviral Effects

Viral infections are contained by innate and adaptive immunity. IFNs, produced during viral (and bacterial) infections are important players in both branches of immunity. The antiviral state established by these pleiotropic cytokines includes IFN-induced metabolic reconfigurations which are in general opposite to the pro-viral metabolic reprogramming described above. The IFN-mediated immune responses and the anti-viral metabolic rewiring processes are closely linked (“immunometabolism”) (Rathmell, 2012; Pollizzi and Powell, 2014). Here we will focus on the IFN-induced metabolic anti-viral and less on the immunological responses. The latter have been more extensively studied in the past (Müller et al., 1994; Stetson and Medzhitov, 2006; Hervas-Stubbs et al., 2011; Hertzog, 2012; Liu et al., 2012; Gessani et al., 2014; McNab et al., 2015), whereas the IFN-triggered metabolic modulations directly affecting viral replication are only beginning to be unraveled (Goodwin et al., 2015; Su et al., 2015; York et al., 2015; Fritsch and Weichhart, 2016; Wu et al., 2016).

### Characteristic Features of IFN-I and IFN-II

IFNs are classified in 3 families: type-I IFNs comprising mainly IFN-α and -β, type-II IFN comprising IFN-γ only, and type-III IFNs (Kotenko et al., 2003; Fay and Pante, 2015; Cohen and Parker, 2016). Since little is known on possible effects of IFN-III on the metabolism of target cells, IFN-III will not be considered further.

IFN-I species are produced by most cell-types. Their synthesis is triggered during viral infections by the interaction of viral nucleic acids with the two ubiquitously expressed cytosolic receptors, RIG-I and MDA5, and several Toll-like receptors (TLR), mainly TLR3, 7, and 9. The latter receptors detect double-stranded RNA (TLR3), single-stranded RNA (TLR7), or unmethylated CpG (TLR9) in the endosome only of certain immune cells, like MPs and DCs. TLR3 is expressed predominantly by conventional DCs (cDCs), whereas TLR 7 and 9 are produced by cDC and pDCs. Especially, pDCs are specialized for IFN-I production in response to TLR7 and TLR9 agonists (Ito et al., 2005; Swiecki and Colonna, 2015) and hence can be activated through an autocrine loop by IFN-I. A major pathway leading to IFN-I production in response to viral (and especially bacterial) infections involves cyclo-di-nucleotides generated by specific dinucleotide cyclases and sensed by the Stimulator of Interferon Genes (STING) (reviewed by Marinho et al., 2017).

Type-II IFN (IFN-γ) is produced predominantly by natural killer cells, natural killer T-cells, TH1, and cytotoxic T-cells upon activation through interaction of T-cell receptors, TCRα,β, with MHC/viral (microbial) antigens (McNab et al., 2015).

Type-I IFNs signal through the IFN-I receptors (IFNAR-1,2) expressed by most cell types, whereas IFN-γ recognizes the receptors FNGR-1,2 expressed mainly by MOs, DCs, and MPs.

The interaction of all IFN types with their cognate receptors leads through activation of several downstream signaling pathways to the expression of large sets of interferon-stimulated genes (ISGs) (Schneider et al., 2014). Each of the IFNs induces unique and partially overlapping sets of ISGs. IFN-I and IFN-III activate similar ISGs albeit with some striking differences (Ioannidis et al., 2013). IFN-γ induced ISGs differ more significantly from those induced by IFN-I and -III (Der et al., 1998; Samarajiwa et al., 2009). Many of these ISGs have direct antiviral effector functions which can target almost any step in a viral life cycle (Schoggins and Rice, 2011; Schoggins et al., 2011). The ISGs comprise also genes encoding enzymes and regulators that are involved in catabolic and anabolic metabolism (Der et al., 1998; de Veer et al., 2001; Mao et al., 2011; Raniga and Liang, 2018).

The fact that downstream signaling pathways activated by IFN/IFNR interactions include, in addition to the canonical Janus kinase/signal transducer and activator of transcription (JAK/STAT) pathway, also the PI3K/Akt and HIF-1 pathways (Horvath, 2004a,b; Kaur et al., 2005, 2008a,b; Glover et al., 2011; Yang et al., 2014) suggests that IFN-induced cell responses also involve metabolic rewiring since the latter signaling pathways, as described above, regulate a variety of basic metabolic pathways (Testa and Tsichlis, 2005; Seo et al., 2011; Semenza, 2013; Xu et al., 2013; Courtnay et al., 2015).

### Type I IFN-Mediated Metabolic Changes

As described above, viruses can reprogram the metabolism of the infected host cell in a pro-viral manner. Inhibition of these pro-viral metabolic events seems to represent part of the anti-viral effects triggered by IFNs. Indeed, there is emerging evidence suggesting that virus-induced type I IFN (especially IFN-α/β) responses affect in particular the energy and lipid metabolism both of which are essential for viral replication (Fritsch and Weichhart, 2016; Ahmed and Cassol, 2017).

IFN-ß induces glucose uptake and glycolytic metabolism through the PI3/Akt pathway in immortalized mouse embryonic fibroblasts (MEF), which seems to be important for the antiviral response during coxsackie virus B3 infection in mice (Burke et al., 2014), possibly by providing the appropriate metabolic conditions for the expression of antiviral ISGs. Induction of aerobic glycolysis and decreased OXPHOS together with IFN-α production has been also observed in human pDCs (vital for antiviral defense) upon stimulation with influenza virus (Bajwa et al., 2016). Interestingly, mouse pDCs respond to IFN-α (when induced by CpGA) with increased FAO and OXPHOS (Wu et al., 2016). This kind of energy supply is also necessary for the defense against lymphocytic choriomeningitis virus (LCMV). The substrates for FAO are produced by *de novo* FAS fueled by glycolysis-derived pyruvate. The peroxisome proliferator-activated receptor α (PPARα) seems to be decisive for regulation of this metabolic modulation in response to IFN-I, but it remains still unclear how PPARα is promoted by IFN-I.

As described above, lipid metabolism (including CL and FA metabolism) is crucial for the replication of most viruses. Hence, inhibition of these pathways contributes to the antiviral action of IFN-I. Inhibition of CL biosynthesis even activates type I IFN production (York et al., 2015; Fessler, 2016).

IFN-I induced by murine cytomegalovirus (MCMV) infection of bone marrow MPs (BMDM) down-regulates the complete sterol pathway resulting in a strong antiviral effect (Blanc et al., 2013). However, while the *de novo* CL biosynthesis is inhibited, CL uptake is stimulated (Boshuizen et al., 2016) which results in a still adequate cellular level of CL essential for cell viability. The decrease in CL biosynthesis appears to be promoted by IFN-I induced through the GAS/cGAMP/STING cascade which is activated by viral DNA (shown with murine γ-herpes virus-68 and HIV-1; York et al., 2015).

Change in FA metabolism has also been observed in non-hematopoietic cells when challenged directly with IFN-I and also upon infection of keratinocytes (using a phocine distemper virus cell line) with LCMV. The observed metabolic change toward FAO and OXPHOS significantly inhibits the virus production, possibly because necessary FAs are catabolized by this metabolic switch. The requirement of *de novo* FAS has been shown for HCMV replication (Munger et al., 2008) and inhibition of FAO in mice diminishes their ability to control a MCMV infection, suggesting that the IFN-I triggered metabolic switch to FAO/OXPHOS is responsible for the antiviral effect caused by IFN-I. Yet, a previous study (Pantel et al., 2014) showed that IFN-I induced in mouse splenic cDCs (stimulated with polyinosinic-polycytidylic acid, polyI:C)—after a transient increase of mitochondrial activity—a switch from OXPHOS to aerobic glycolysis. This metabolic switch requires the up-regulation of HIF-1 which is also necessary for survival and immunogenicity of the DCs in mice. It remains an open question whether the obviously divergent results of the two latter studies are due to the different sources of the used DCs (bone marrow vs. spleen DCs) or the different stimuli used for IFN-α production in the DCs (stimulation by CpGA vs. polyI:C). There seems to be, however a general difference in the metabolic modulation of pDCs and cDCs, since even splenic cDCs exhibit upon stimulation by CpGA an early glycolytic response before OXPHOS increases (Wu et al., 2016).

IFN-I induced by polyI:C in bone marrow-derived MPs, BMDMs, and DCs, also promotes up-regulation of cholesterol-25 hydroxylase (CH25H), an ISG product that inhibits viral entry (Liu et al., 2013; Chen et al., 2014; Raniga and Liang, 2018). CH25H converts CL to 25-hydroxycholesterol. This metabolite directly inhibits (as shown in various cell types) growth of different enveloped viruses that can cause persistent (e.g., VSV, HSV, HIV) or acute infections (e.g., Ebola virus, Rift Valley fever virus), probably by blocking cell/virus membrane fusion during viral entry (Liu et al., 2013).

Together, virus-induced type-I IFN production causes a variety of metabolic changes in different target cells (especially DCs and MPs) that contribute to the antiviral effect triggered by these cytokines. These metabolic modulations, affecting in particular the energy and lipid metabolism, may—in addition to known effects on antiviral immune responses—directly inhibit viral replication.

#### Metabolic Changes Triggered by IFN-γ

Type-II IFN (IFN-γ) interacts also especially with DCs and MPs. The antiviral effect of IFN-γ is mainly attributed to the enhanced expression of specific ISGs and to inflammatory responses (Hu and Ivashkiv, 2009; Qiao et al., 2013). Treatment of MPs with lipopolysaccharide (LPS) and IFN-γ leads to the classical activation of MPs (M1-polarization) which is characterized by the switch from OXPHOS (occuring in resting MPs) to induced glucose uptake, aerobic glycolysis with lactate production, enhanced PPP and decreased TCA activities (Kelly and O'Neill, 2015). However, this switch is also induced by LPS alone (Krawczyk et al., 2010; O'Neill, 2014), indicating that IFN-γ plays in this context mainly a co-stimulatory role, probably by enhancing the expression of the LPS receptors CD14 and TLR4 (Lappin et al., 2016).

Recently, Su et al. (2015) showed, however, that IFN-γ may directly reprogram the metabolism in human MPs by partially suppressing mTORC1, leading to down-regulation of the translation of many mRNAs, but also to enhanced translation of specific mRNAs involved in anti-viral effects. IFN-γ (and to a lesser extent type I IFNs) strongly induces indoleamine-2,3-dioxygenase 1 (IDO) in MPs and pDCs (Yoshida et al., 1981; Mellor and Munn, 2004; Raniga and Liang, 2018). IDO degrades Trp to kynurenine (Kyn) and Kyn derivatives thereby depleting the intracellular Trp pool. This may have a dual effect on viral replication: (a) The increased level of cellular Kyn can cause immunosuppression thus favoring viral infections. (b) The Trp depletion may reduce the metabolic activity of the host cell which will inhibit viral replication (Schmidt and Schultze, 2014). Indeed, replication of several viruses, including HCMV, vaccinia virus, HSV, MV, and hepatitis B virus have been shown to be highly sensitive to the Trp depletion (Bodaghi et al., 1999; Adams et al., 2004; Obojes et al., 2005; Terajima and Leporati, 2005; Mao et al., 2011). The IDO-catalyzed cellular Trp depletion could also explain the IFN-γ-mediated suppression of the mTORC1 activity (Su et al., 2015): mTORC1 is activated by elevated levels of amino acids, especially Leu but also Trp (Hara et al., 1998; Su et al., 2015). Depletion of intracellular Trp may therefore suppress mTORC1 activation thereby diminishing the translation of multiple proteins including those essential for viral proliferation (Su et al., 2015; Kroczynska et al., 2016).

IFN-γ can also repress the expression of the NAD^+^-dependent deacetylase SIRT-1 (Li et al., 2012) which is a critical coordinator of cellular metabolism acting primarily by deacetylation. A decreased level of SIRT-1 disrupts the expression of several metabolic genes which may also contribute to the antiviral effect exerted by IFN-γ.

In summary, type I as well as type II IFNs directly induce in DCs and MPs (often initial host cells in viral infections), in addition to the known antiviral immune responses triggered by inflammatory cytokines, metabolic changes that inhibit viral replication more directly and thus contribute to the IFN-mediated antiviral host responses. These antiviral metabolic modulations are opposed to the above described proviral metabolic reprogramming of host cells (Tables 1, 2). It remains an open question whether these apparently opposite metabolic events may occur within the same virus-infected host cells in a chronological order during infection or in different subsets of the host cell population.

## Metabolic Changes Caused by IBP/Host Cell Interactions

Compared to the above described information on virus-induced metabolic reprogramming of host cells much less is known on metabolic modulations caused by infection with IBPs which—similar to viruses—replicate in (often the same) host cells.

As described, viruses modulate catabolic and anabolic pathways of their host cells predominantly by interaction of specific viral components with enzymes and signaling factors, especially oncogenes, and tumor suppressors, controlling metabolic pathways (see Figure 4 and Tables 1, 2).

Most of the studies conducted on IBP replication have used as host cells different established cell lines, including MO- and MP-like cell lines (e.g., J774A.1, P388.D1, RAW264.7, THP-1, U-937) as well as epithelial and fibroblast cell lines (e.g., Caco-2, HeLa, Hep-2, HEK293, MDCK, NIH3T3, and others). As a major result, the studies show that these host cells allow highly efficient intracellular replication of most IBPs (Eisenreich et al., 2015, 2017). Most of these cell lines perform already in the un-infected state a highly activated metabolism, in most cases caused by the permanent activation of oncogenes (e.g., Myc in J774 MPs) or the inactivation of tumor suppressors (e.g., p53 in Caco-2, HeLa, U-937, THP-1) (Scheffner et al., 1991; Sugimoto et al., 1992; Berglind et al., 2008). This host cell metabolism which is characterized in general by enhanced glucose uptake, aerobic glycolysis, increased PPP activity, eventually enhanced glutaminolysis, and increased anabolic activities apparently meets the metabolic requirements of many IBPs for efficient intracellular replication and proliferation (Fuchs et al., 2012; Eisenreich et al., 2013; Kentner et al., 2014; Escoll and Buchrieser, 2018). In accord with this assumption, little change in the metabolic fluxes of Caco-2 cells is observed upon infection with IBPs, like *Listeria monocytogenes, Salmonella* Typhimurium, or enteroinvasive *Escherichia coli* (Götz et al., 2010). These data indicate that no further metabolic reprogramming in these host cells is necessary to satisfy the nutritional needs of the IBPs for efficient intracellular growth. Yet, there are also exceptions, e.g., *Chlamydia pneumoniae* infection of Hep-2 cells leads to additional stabilization of HIF-1α resulting in further enhanced glucose uptake during the early phase of infection which favors bacterial proliferation (Rupp et al., 2007); some other examples will be discussed below. These few examples already indicate that established cell lines are of limited value for studies intended to unravel the metabolic host cell responses upon *in vivo* infection by IBPs.

**Figure 6:**
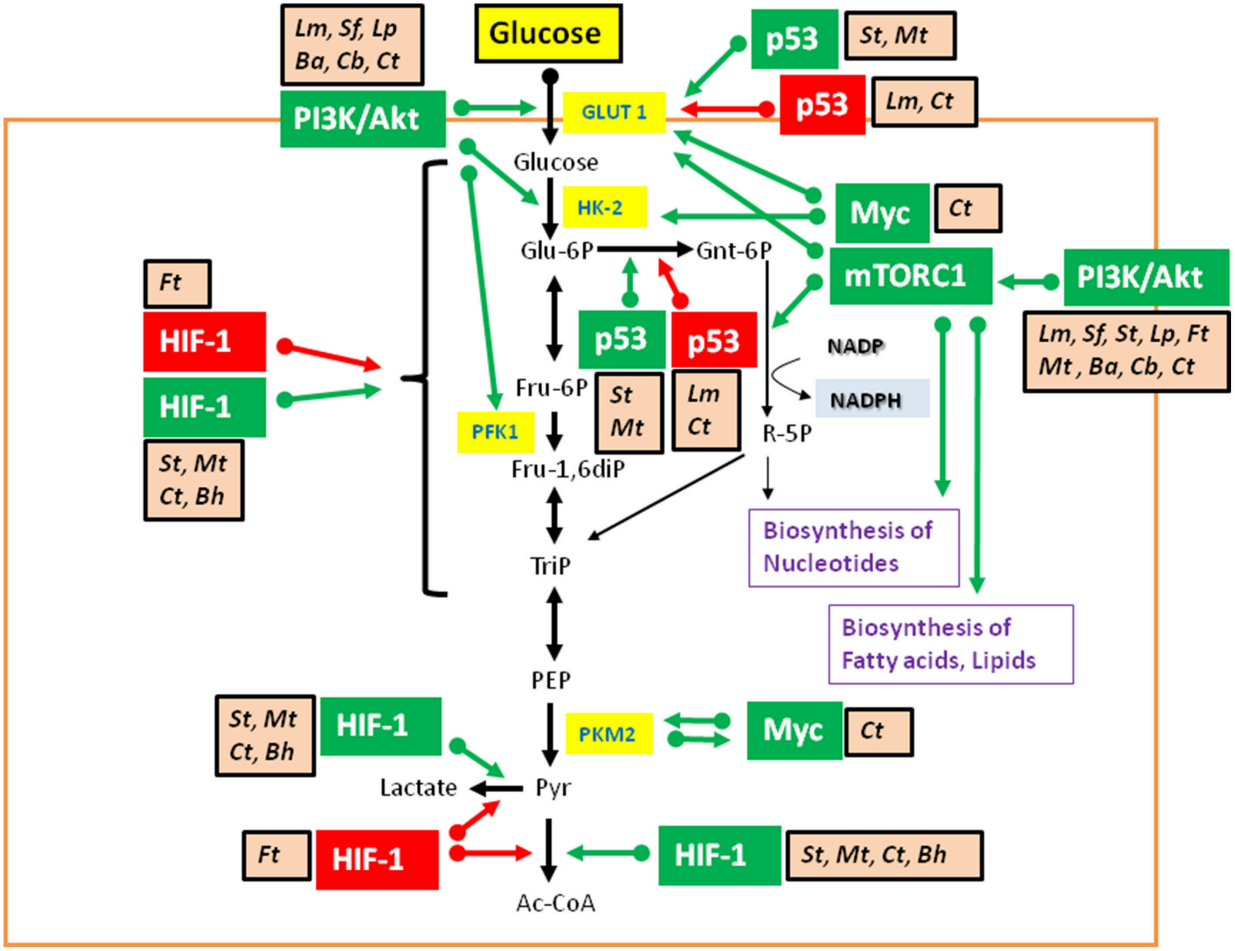
Intracellular bacterial pathogens (IBPs) influence the activity of central metabolic regulators of their host cells. Bacterial factors activate components of the PI3K/Akt/mTOR cascade and Myc, or alter the concentration and/or activity of p53 and HIF-1 (see text and Table 3 for details). Most of these interactions lead to enhanced glucose uptake, increased aerobic glycolysis and enhanced PPP activity as well as to activation of anabolic pathways in the infected host cells. Activation of Myc by some IBPs also enhances Gln uptake and glutaminolysis. *Lm, Listeria monocytogenes*; *Sf*, *Shigella flexneri*; *St, Salmonella enterica*; *Lp, Legionella pneumophila*; *Mt, Mycobacterium tuberculosis*; *Ba, Brucella abortus*; *Bh, Bartonella henselae*; *Cb, Coxiella burnettii*; *Ct, Chlamydia tracho*matis; *Ft, Francisella tularensis*. For other abbreviations, see Figures 1, 2.

Studies on metabolic responses induced in primary cells or animal models upon infection by IBPs are rare. These studies (mostly performed in other, mainly immunological contexts) frequently show (similar to virus infections): (a) interactions with components of the PI3K/Akt signaling pathway (Asare and Kwaik, 2010; Cremer et al., 2011; Huang, 2014; Jiang et al., 2014; Subbarayal et al., 2015; Bonnet and Tran Van Nhieu, 2016) as well as inhibition or promotion of phosphoinositide synthesis (Pizarro-Cerda and Cossart, 2004), (b) reduced p53 gene expression, inhibition of the activity of p53 protein, or induction of p53 degradation (Bergounioux et al., 2012; Siegl et al., 2014; Siegl and Rudel, 2015; Zaika et al., 2015), and (c) activation of HIF-1 (mainly by stabilization of HIF-1α) (Hartmann et al., 2008; Werth et al., 2010;Devraj et al., 2017) (Figure 6).

These IBP-triggered host cell processes may, however, also lead to reprogramming of the host cell metabolism (similarly as in viral infections—see Figure 4 and Tables 1, 2). Yet, the expected metabolic consequences have rarely been analyzed and little is known on the bacterial factors that trigger these IBP-mediated processes (Escoll and Buchrieser, 2018).

In the following, we will discuss metabolic responses of host cells triggered by isolated bacterial components and intact IBPs, putting special emphasis on studies that use primary cells, organ(oid)s, or animals as infection models.

### Different Metabolic Programs Are Triggered by the Interaction of Isolated IBP Components and Intact IBPs With Target Cells

Interesting metabolic studies have been performed in the context of immunometabolism which analyze the effect of isolated bacterial components (mainly pathogen-associated molecular patterns, PAMPs, on the metabolism of resting primary MPs). These studies are worth mentioning here, since they demonstrate the problem of comparing such metabolic cell responses with those obtained by infection with intact live IBPs. As an example, we will discuss the interaction of resting MPs with LPS which leads by binding to CD14/TLR4 to the “classical activation” of the MPs (Biswas and Mantovani, 2012). The resulting “M1 phenotype” is highlighted by a switch from OXPHOS (characteristic for resting MPs) to induced glucose uptake, aerobic glycolysis combined with lactate production, enhanced PPP and decreased TCA activities. This metabolic program causes induced synthesis of inflammatory cytokines as well as nitrogen and oxygen radicals (Kelly and O'Neill, 2015) and is highly adverse for replication of most IBPs (Eisele et al., 2013; Xavier et al., 2013; Price and Vance, 2014).

The “alternative activation” of MPs (induced e.g., by IL-4) leads to the anti-inflammatory M2 phenotype which is characterized by enhanced FAO, OXPHOS, and increased intracellular levels of unconsumed glucose. This host cell metabolism seems to be favorable for IBP replication. It has been suggested that M2 MPs could generally represent a suitable metabolic state for IBPs (Xavier et al., 2013; Buchacher et al., 2015).

However, the idea that M2-MPs are allies and M1-MPs adversaries for IBPs appears to be too simple. As mentioned above, the metabolism of MO- or MP-derived cell lines, like J774A.1, P388.D1, RAW264.7, or U-937 is mainly activated by constitutively expressed oncogenes or inactivated tumor suppressors and is distinct from that of M1 or M2 MPs (Götz et al., 2010; Gillmaier et al., 2012; Mehlitz et al., 2017). Yet, it provides excellent conditions for IBP replication. Indeed, metabolic programs can be also induced in primary MPs that are different from those of the M1 or M2 MPs (Murray and Wynn, 2011; Guilliams et al., 2014; Murray et al., 2014).

Although most IBPs are LPS-producers, and their purified LPS most likely triggers a M1 phenotype when added to primary MPs, most intact IBPs reprogram the metabolism of MPs differently and beneficially for their replication, e.g., by stimulation of growth hormone receptors, activation of corresponding signaling pathways, activation of oncogenes or inactivation of tumor suppressors by specific IBP effectors. In addition, IBPs may counteract antimicrobial responses caused by an adverse cell metabolism and hence even overcome the hostile metabolic background of M1-MPs (see below).

### Metabolic Reprogramming of Host Cells Upon IBP Infections Using Primary Cells and Animal Models

IBPs are able to replicate within their host cells in IBP-specific vacuoles, in the cytosol or even in both compartments (Knodler, 2015). The replication rate of IBPs residing in the cytosol is in general higher than that of IBPs replicating in membrane-surrounded vacuoles which suggests an easier access to essential nutrients by cytosolic IBPs compared to vacuolar IBPs. This can, however, also lead to a faster exhaustion of essential nutrients in the host cells by cytosolic IBPs with possible negative effects for the infected cells (e.g., induction of autophagy, production of reactive oxygen species and other antimicrobial cell responses). To overcome these diverse effects, IBPs have developed various strategies, including reprogramming of the host cell metabolism (Figure 6) that are well-adapted to the specific metabolic needs of the individual IBP in its cellular niche. In the following we summarize what is known on this intriguing, but still poorly studied aspect in the intracellular life cycle of IBPs.

#### IBPs Replicating in the Cytosol

*Listeria monocytogenes* significantly activate the glycolytic pathway and the initial part of the TCA upon infection of BMDMs (Gillmaier et al., 2012; Eisenreich et al., 2017). Yet, only a small percentage of the BMDM population is infected with high numbers of bacteria while most MPs contain few or no bacteria. This finding suggests that only a subpopulation of the primary MPs is adequately activated—possibly by the interaction of specific listerial factor(s) with a suitable MP receptor (Eisenreich et al., 2017)—and thereby prepared for efficient intracellular replication of *L. monocytogenes*. This assumption is supported by the fact that J774 MP-like cells (a cell line which carries out a Myc-activated metabolism) are infected by *L. monocytogenes* at a much higher rate and readily support efficient listerial replication (Eisenreich et al., 2017).

Down-regulation of p53 and p53 knockout mice results in increased intracellular replication of *L. monocytogenes* whereas the intracellular replication is inhibited by overexpression of p53 (Wang et al., 2016). Reduction of p53 leads, similar as Myc activation, to induced glucose uptake, subsequent aerobic glycolysis and reduced OXPHOS. This metabolic host cell program apparently provides favorable conditions for intracellular *L. monocytogenes* proliferation.

In this context, it should be noted that the metabolic flux (including the glycolytic flux) is down-regulated in J774 cells upon *L. monocytogenes* infection in contrast to its up-regulation in BMDMs. However, the metabolism of the J774 cells is already highly activated (due to the enhanced Myc activity) and the infection may generate a nutrient stress on these cells. This apparent discrepancy between the metabolic host cell responses of primary and cancer cells upon infection by the same IBP shows once again that metabolic results obtained with IBP-infected established cell lines should be considered with great caution.

Members of the genus *Shigella* (mainly *S. dysenteriae, S. flexneri*, and *S. sonnei*) and the closely related enteroinvasive *E. coli* (EIEC) are cytosolic IBPs, infecting preferentially primates. Hence, primary cells and *bona fide* animal models are difficult to establish (Anderson et al., 2016; Killackey et al., 2016). Indeed, most studies dealing with metabolic host cell responses triggered by *S. flexneri* infection are performed with human intestinal cancer cell lines (HeLa, Henle 407, and Caco-2) or with primary human umbilical vein endothelial cells (HUVECs) of undefined metabolic status (Tattoli et al., 2012; Pieper et al., 2013; Kentner et al., 2014; Waligora et al., 2014). The used metabolically activated cancer host cells are apparently in a metabolic state that is highly suitable for supporting the intracellular bipartite metabolism of *S. flexneri*. This is based on pyruvate (or lactate) as energy source (Kentner et al., 2014) and glucose (or glucose-6-phosphate, G6P) for performing the indispensable anabolic activities (Götz and Goebel, 2010), especially biosynthesis of components of the cell envelope (see above). It is therefore not surprising that *Shigella* infection does not further induce the already highly activated glycolytic pathway of these host cells, but since pyruvate is now consumed by the intracellular *Shigella* as energy source, the infected host cells excrete acetate instead of lactate (Kentner et al., 2014). Due to the metabolic stress exerted on the host cells by the infection, the basic metabolic pathways of the infected cells are again slightly inhibited (Götz et al., 2010; Gillmaier et al., 2012; Kentner et al., 2014). Specifically, starvation of the infected cells due to amino acid consumption by the IBPs leads to inhibition of mTORC1 and induction of integrated stress response pathway (Tattoli et al., 2012). However, these studies do not answer the crucial question whether *Shigella* infects *in vivo* host cells that are metabolically already prepared for supporting intracellular *Shigella* replication or whether *Shigella* is able to actively reprogram (or activate) the metabolism of (even metabolically quiescent) host cells to a state that allows intracellular replication of *Shigella*.

In this context, the observation that *Shigella* targets cells of the colonic crypts at early time points (Arena et al., 2015) in the guinea pig model (Shim et al., 2007) is of special interest. Crypts harbor proliferating Lgr5^+^ stem cells essential for the regeneration of differentiated intestinal epithelial cells and Paneth cells. In the murine model, these two cell types at the base of the crypt are metabolically highly active (Stringari et al., 2012; Rodriguez-Colman et al., 2017). While Paneth cells show an enhanced glycolytic phenotype and secrete lactate, the metabolism of the Lgr5^+^ stem cells is based on increased OXPHOS which is supported by the secreted lactate of the Paneth cells. The lactate is apparently taken up by the Lgr5+ cells and converted to pyruvate (Rodriguez-Colman et al., 2017). This Lgr5^+^ cell population could thus represent suitable early host cells for *Shigella*.

*Francisella tularensis* is able to infect numerous mammalian cell types and causes plague-like illness in lagomorphs. MPs represent the main host cells where these Gram-negative bacteria efficiently replicate in the cytosol (Celli and Zahrt, 2013). These host cells are also mainly responsible for *in vivo* dissemination. ^13^C-Isotopolog profiling studies with human-pathogenic and non-pathogenic *F. tularensis* subspecies growing in culture media reveal glucose as the most efficient carbon substrate (Brissac et al., 2015; Chen et al., 2017); glycerol and pyruvate (but not Ser) could also serve as carbon source for growth of *F. tularensis*, however with substantially lower rate. *In vivo* replication of *F. tularensis* (in mice and BMDM) apparently relies on several host-derived carbon sources, including a complex set of amino acids, especially Cys (Alkhuder et al., 2009). Two amino acid transporters involved in uptake of Glu and Asn, respectively, have been shown to be essential for the cytosolic life cycle (Barel et al., 2015). In the presence of pyruvate as major substrate (under glucose limiting conditions), reduced intracellular replication is observed which depends on gluconeogenesis (Brissac et al., 2015; Ziveri et al., 2017). Thus, *F. tularensis* seems to also follow for efficient replication within host cells—similar to the other cytosolic IBP—a bipartite metabolism with pyruvate, glycerol and Cys (or possibly also other amino acids) as major energy source, supplemented by glucose as essential substrate for the indispensable bacterial biosyntheses (mainly those yielding cell envelope components and nucleotides).

Extensive metabolic reprogramming of primary murine BMDMs (as host cells) is observed when infected with *F. tularensis*. Apparently, alternative activation of MPs (M2-polarized) does not occur upon infection. Elimination of arginase 1 leading to enhanced NO production, also does not significantly alter the intracellular replication efficiency of *F. tularensis* (Griffin et al., 2013). Rather, *F. tularensis* down-regulates HIF-1α in the primary MPs and thus prevents the shift to aerobic glycolysis (characteristic for M1-polarized MPs). This metabolic reprogramming of the host cells is required for optimal intracellular replication of *F. tularensis*. The *Francisella* capsule appears to be involved in this process (Wyatt et al., 2016). Furthermore, induced Atg5-independent autophagy in *F. tularensis* infected cells supports intracellular *F. tularensis* replication, probably by providing additional nutrients (Steele et al., 2013). *Francisella* itself is protected against autophagic killing by surface polysaccharides (Case et al., 2014).

In contast to *S. flexneri, L. monocytogenes*, and *F. tularensis, Rickettsia prowazekii* is an obligate intracytosolic pathogen that has a highly reduced repertoire of catabolic and anabolic pathways. The genome of this Gram-negative bacterium lacks all genes for glycolysis/gluconeogenesis and for both arms of the PPP, but contains the entire gene set encoding the enzymes of the TCA. ATP is in part transported via specific ATP translocases from the host cell (Walker and Yu, 2005), but once the bacteria have used up the host ATP, they may initiate ATP generation by OXPHOS via its own ETC using NADH/H^+^ (generated in the TCA by oxidation of α-KG derived from host-imported Glu) (Weiss et al., 1989; Renesto et al., 2005), of OAA (derived from host aspartate) or of pyruvate (derived from host Ser) (Austin et al., 1987). These amino acids serve as essential energy sources for intracellular growth of *R. prowazekii*. ATP is possibly also generated via substrate phosphorylation by converting pyruvate to acetate; the rickettsial genome contains the genes for this ATP generating pathway (Renesto et al., 2005; Walker and Yu, 2005).

Although *R. prowazekii* lacks most anabolic pathways (Andersson et al., 1998; Walker and Yu, 2005), it produces some anabolic metabolites that cannot be provided by the host cell, most notably mDAP essential for its PG biosynthesis and 2-keto-3-deoxyoctulonic acid essential for its LPS synthesis. Furthermore, *R. prowazekii* utilizes at least two triose phosphate acquisition pathways. Glycerol-3-phosphate is directly transported and incorporated into phospholipids (Frohlich et al., 2010; Frohlich and Audia, 2013) and, in addition, the gene for a GlpT-homologous glycerol-3-phosphate transporter has also been identified in the *R. prowazekii* genome (RP054).

The high dependence of this obligate intracytosolic pathogen on nutrient and metabolite supply from the host cell is also reflected by the numerous transport systems which have been identified in the genome of *R. prowazekii* (Andersson et al., 1998; Walker and Yu, 2005). This dependence on host metabolites most likely requires massive reprogramming of the host cell metabolism upon infection by this pathogen. However, to our knowledge nothing is known until now concerning this important aspect.

Taken together, substantial progress has been made regarding the intracellular metabolism of these cytosolic IBPs which shows remarkable similarities although the metabolic capabilities of the four described IBPs differ very significantly: All four IBPs described seem to rely for its intracellular replication on a bipartite metabolism using a host-derived energy-rich C_3_-metabolite (which is less significant for the host cell than glucose) as major energy source. Depending on the IBP, this is either directly pyruvate or a metabolite that can be converted to pyruvate, such as lactate, glycerol, Ser, and Cys. Although the conversion of pyruvate to acetate as major ATP-generating pathway has been experimentally shown so far only for *Shigella* (Kentner et al., 2014), it is striking that all four cytosolic IBPs possess this ATP-generating pathway as well as the ability to convert Ser to pyruvate. It is also remarkable that these IBPs, including the metabolic generalist *Shigella* (Götz et al., 2010; Kentner et al., 2014), import many anabolic metabolites (in particular amino acids) from the host cell and seem to limit their anabolic activities mainly on those products that cannot be provided even by a nutrient-rich host cell. These include in particular components necessary for the formation of the IBP-specific components of the cell envelop (PG, LPS), and of the ETC, e.g., menaquinone (Stritzker et al., 2004). These well-adapted cytosolic IBPs have also developed mechanism to resist host cell autophagy (in contrast to most other microbes that may enter the host cell cytosol) and can even use nutrients that are released by autolysis of host macromolecules or storage structures for their own metabolism (Uchiyama, 2012; Steele et al., 2015). However, we still know little on the precise status of primary target host cells that are infected by these IBPs *in vivo* (especially in humans) and even less on the metabolic reprogramming which these cells undergo during infection.

#### IBPs Replicating in Specialized Vacuoles

The majority of the human IBPs replicate within their host cells in membrane-bound compartments (Kumar and Valdivia, 2009). The most extensively investigated members of this group belong to the genera *Salmonella, Legionella, Mycobacterium, Brucella, Bartonella, Coxiella*, and *Chlamydia*. The biogenesis of these PCVs is complex. For details, see recent reviews by Sherwood and Roy (2013) and Creasey and Isberg (2014). The IBP-specific modification of the vacuolar niche, aimed to enable survival and replication of the respective IBP, requires specific bacterial proteins and lipids and involves also the subversion of host cell secretory pathways. The necessary IBP-specific membrane rearrangements and *de novo* synthesis of lipids triggered by the infection in the host cell will be outlined in more detail below.

In general, intravacuolar IBPs obtain host cell nutrients via host- or pathogen-derived transporters inserted in the vacuolar membrane or by exchange with endocytic vesicles (Abu Kwaik and Bumann, 2015; Liss and Hensel, 2015). By these routes the IBPs may import glucose from the host cells cytosol in a more controlled manner or from the extracellular environment through exocytic vesicle transport. The latter type of glucose uptake will less deteriorate the glucose balance (homeostasis) of the host cell than the direct glucose consumption from the host cell's cytosol by the cytosolic IBPs. Indeed, when *Salmonella enterica*, which normally lives in the *Salmonella*-containing vacuole (SCV), enters the host cell cytosol it replicates much faster than in the SCV; but in this state the *Salmonella*-infected host cell is killed more quickly via pyroptosis than when the bacteria remain in the SCV state (Knodler, 2015).

The use of glucose (or G6P and other glucose-generating substrates, e.g., glycogen) as essential nutrient for intracellular replication of this group of IBPs has been reported for *Salmonella* (Bowden et al., 2009; Eisele et al., 2013), *Brucella* (Xavier et al., 2013), *Legionella pneumophila* (Manske and Hilbi, 2014; Eisenreich and Heuner, 2016), *C. trachomatis* (Gehre et al., 2016), *Brucella abortus* (Xavier et al., 2013), *Coxiella burnetii* (Vallejo Esquerra et al., 2017), and *Mcobacterium tuberculosis* (Mt) (Shi et al., 2015). But all these IBPs can use (similar to the cytosolic IBPs) in addition to glucose one or more other host-derived metabolites, such as lactate, pyruvate, glycerol, malate, Fas, or certain amino acids. These substrates are nutrients which probably serve preferentially as an energy source (Steeb et al., 2013; Häuslein et al., 2016; Mehlitz et al., 2017). Since these latter nutrients are also gluconeogenic substrates it is not surprising that gluconeogenesis may occur in these IBPs (all of which encode the necessary enzymes) when glucose as essential component for the biosynthesis of cell surface structures runs short.

The majority of these vacuolar IBPs possesses (again similar to the cytosolic IBPs) the genetic potential to convert pyruvate to acetate via acetyl-phosphate generating ATP by substrate phosphorylation. Only the two obligate IBPs, *Coxiella* and *Chlamydia*, are apparently unable to carry out this energy-generating reaction. The acidic conditions (pH 4.5–4.7) (Omsland et al., 2011) required for optimal growth of *Coxiella* are probably unfavorable for the ATP generating acetate kinase reaction, since acetyl-phosphate may be hydrolyzed to acetate and phosphate. Human pathogenic *Chlamydia*, on the other hand, have limited options to obtain pyruvate and hence pyruvate may not be a suitable substrate for ATP production. But these two obligate IBPs harbor two ATP/ADP translocases that allow ATP uptake from the host cell (Fisher et al., 2013). Together, it seems that this group of IBPs also follows—for performing an efficient basic carbon and energy metabolism within their host cell niches—the strategy of “bipartite metabolism” (see above), although the routes for obtaining the necessary nutrients are different from those of cytosolic IBPs.

Like the cytosolic IBPs, the vacuolar IBPs consume substantial amounts of host cell-derived nutrients for efficient replication. Again, many established cell lines are excellent host cells for the intracellular replication of this group of IBPs. In *in vivo* infections, MOs/MPs are also often the primary host cells for vacuolar IBPs. The PI3K/Akt/mTOR pathway, p53 and HIF-1 which are important regulatory factors in the host cell metabolism (see above) are again frequent targets in these host cells when infected with members of this group of IBPs as outlined in more detail below (see also Figure 6 and Table 3).

**Table 3:** Intracellular bacterial pathogens: Interactions of bacterial factors and host cell targets expected to cause metabolic reprogramming of the IBP-infected cells.

**Intracellular bacterial pathogen (IBP)**	**Interacting bacterial component**	**Host cell target**	**Used host cells and references**
**VACUOLAR IBPs**			
*Salmonella typhimurium*	SopB	PI3K/Akt/mTOR (+)	HeLa cells (Cooper et al., 2011)
	SopB	Class II PI3-kinases activate Akt	HeLa and mouse embryonic fibroblast cells (MEFs) (Roppenser et al., 2013)
	SP-2 effector(s)	FAK/Akt/mTOR (+)	Peritoneal MPs (PEMs) (Owen et al., 2014)
	Salmochelin	HIF-1 (+)	HeLa cells, HMEC (Hartmann et al., 2008)
	Unknown	p53 (+)	HCT116, IEC-18 cells, and MEFs (Wu et al., 2010)
*Legionella pneumophila*	Dot/Icm effector protein(s)	PI3K/Akt/mTOR (+)	Mouse BMMs (Abshire et al., 2016)
	Lgt effector family	mTORC1 (+)	(Escoll and Buchrieser, 2018)
	SidE effector family	mTORC1 (–)	(De Leon et al., 2017)
*Mycobacterium tuberculosis*	Unknown	PI3K/Akt/mTOR (+)	PBMCs (Lachmandas et al., 2016)
	Unknown	HIF-1 (+)	C57BL/6 mice (Shi et al., 2015)
	Unknown	p53 (+)	Blood human MOs (Galietti et al., 2001)
*Brucella abortus*	Unknown	Warburg shift	THP-1 cells (Czyz et al., 2017)
*Chlamydia trachomatis*	Unknown	PDPK1, Myc, HK-2 (+)	HeLa cells, fallopian tube primary epithelial organoids (Al-Zeer et al., 2017)
	Unknown	HIF-1 (+)	HeLa cells (Sharma et al., 2011)
	Unknown	p53 (–)	HUVEC cells (Siegl et al., 2014)
*Coxiella burnetii*	Unknown	Akt (+)	Primary human alveolar MPs (Hussain and Voth, 2012)
**CYTOSOLIC IBPs**			
*Shigella flexneri*	OspB	mTOR (+)	HeLa, mouse embryonic fibroblasts (MEFs) (Lu et al., 2015)
	Unknown	Akt (+)	HeLa cells
		mTOR (–)	(Tattoli et al., 2012)
*Listeria monocytogenes*	InlB	PI3K/Akt (+)	LS174T and Jar cell lines (Gessain et al., 2015)
	InlB	Met-dependent phosphorylation of mTOR (+)	HeLa cells (Bhalla et al., 2017)
*Francisella tularensis*	Unknown	PI3K (+), mTOR (+)	Peritoneal MPs from C57BL/6 mice (Edwards et al., 2013)
	Capsule	HIF-1 (–)	BMDM of C57BL/6 mice (Wyatt et al., 2016)

*The host cells used in the studies are also indicated*.

*Salmonella enterica*—in contrast to the cytosolically replicating enterobacterium *Shigella* (see above)—leads to activation of p53 upon infection of intestinal epithelial cells. These cells lacking p53 are less susceptible to *Salmonella* infection (Wu et al., 2010). Activated p53 is expected to rather silence than activate the host cell metabolism. However, one should also keep in mind, that p53, generally activated in cellular stress controls, is also involved—besides controlling the above mentioned metabolic pathways—in other cellular key processes, like apoptosis, autophagy and cell proliferation through inhibition of Akt and mTOR (Liang et al., 2013). Hence, changes in the expression or activation of p53 by IBP infection also influence apoptosis, autophagy, and the Akt/mTOR pathway in infected cells. These changes may have a strong impact on the survival and nutrient availability of the host cell which in turn can influence the replication of the IBPs.

*Salmonella* uses the type 3 effector protein SopB to manipulate multiple host kinases (besides PI3K) for activation of Akt which optimizes *Salmonella* replication in host cells (Roppenser et al., 2013). However, the likely contribution of this Akt activation to reprogramming of the host cell metabolism has not yet been determined. *S. enterica* also activates HIF-1 by its siderophore salmochelin independent of cellular hypoxia in human epithelia and endothelia by inhibition of prolylhydroxylase (Hartmann et al., 2008). HIF-1 activation may also lead to increased glucose uptake and induced aerobic glycolysis in the infected cells (Semenza, 2011).

*Legionella pneumophila* is able to enter suitable host cells (especially MPs) and activates PI3K/Akt signaling (Tachado et al., 2008). Within host cells, *L. pneumophila* forms the *Legionella*-containing vacuole (LCV), where it efficiently replicates before it exits the host cell by lysis. The LCV formation requires membrane biogenesis with sustained supply of host lipids during expansion (Abshire et al., 2016). In *Legionella*-infected MPs the lipid supply involves uptake of lipids from serum as well as *de novo* lipogenesis. The latter is controlled via mTOR by SREBP-1 and−2.

Throughout its intracellular infection cycle, *L. pneumophila* replication requires sustained mTOR signaling (Abshire et al., 2016; Ivanov, 2017) by a process that involves PI3K activation at the host cell side and one or more Dot/Icm (yet unknown) effector proteins at the bacterial side. Indeed, inhibition of the PI3K/mTOR/SREBP-1/2 axis blocks LCV expansion and leads to death of the infected MPs, indicating that mTOR-dependent lipogenesis in the host cells is necessary for optimal intracellular replication of *L. pneumophila*. Furthermore, it has been demonstrated that *L. pneumophila* translocates the effector protein sphingosine-1-phosphate lyase (LpSpl) which targets the host cell sphingosine biosynthesis (causing disruption of sphingolipid formation) and inhibits autophagy (Rolando et al., 2016a,b). LpSpl appears to be also required for efficient infection of A/J mice, indicating that this effector protein represents a kind of metabolic virulence factor (Rolando et al., 2016b).

Like most IBPs, *L. pneumophila* requires also amino acids from the host cell for intracellular growth (Eylert et al., 2010). Two effector families, Lgt and SidE, were shown to interact with mTORC1, thereby commonly inhibiting mTORC1 activity and hence host cell protein synthesis (De Leon et al., 2017). This concerted action of the two effector families (and possibly still other effector proteins interfering with mTORC1) may liberate host amino acids for the consumption by *L. pneumophila*. As shown in Table 3, mTORC1 appears to be a frequent target in IBP infections.

It should be noted, however, that the above data are obtained by *L. pneumophila* infection of cell lines (J774, HeLa, HEK293, and others) which already show a highly activated carbon metabolism and hence do not allow conclusions (besides the described enhanced lipogenesis and the mTORC1 inhibition) concerning the complete metabolic reprogramming of host cells by *L. pneumophila in vivo* infection.

More recently, it has been reported (Escoll et al., 2017) that *L. pneumophila* induces a metabolic reprogramming in human primary MPs by MitF-mediated mitochondrial fragmentation (MitF is a T4SS effector protein of *L. pneumophila*). This fragmentation apparently results in a Warburg-like metabolism in the infected MPs and supports replication of *Legionella*. Mitochondrial fragmentation (causing inhibition of OXPHOS) has been already postulated by Warburg as a cause for the switch to aerobic glycolysis in cancer cells (Warburg et al., 1927). However, whether this really explains the metabolic adaptation of the host cell to *L. pneumophila* infection still remains an open question.

Mt infection of murine lung tissue leads to increased levels of HIF-1α within MPs and T-cells present in granulomatous lesions. In accord with this HIF-1 activation, transcriptomic profiling and confocal imaging show up-regulation of key glycolytic enzymes, GLUTs and MCT4 transporter (essential for lactate secretion), as well as down-regulation of TCA and OXPHOS (Shi et al., 2015), i.e., the typical “Warburg-effect.” Tuberculous granulomas are agglomerations of infected and uninfected immune cells, including MPs, neutrophils, T-cells, and other immune cells. The above metabolic results represent average values deriving from the entire infected and uninfected MP (and T-cell) population. It is therefore difficult to deduce from these data which metabolic program supports intracellular mycobacterial replication or persistence in potential host cells within the granulomas. A similar metabolic reprogramming toward aerobic glycolysis has also been observed in human peripheral blood mononuclear cells (PBMCs) (Lachmandas et al., 2016) and human alveolar MPs (Gleeson et al., 2016) after infection by live Mt—but also by Mt lysates. This metabolic switch is TLR2 dependent and mediated through activation of the Akt/mTOR pathway (Lachmandas et al., 2016). However, these studies were intended to show that this metabolic switch is necessary for mounting efficient immune responses to Mt, rather than demonstrating that this metabolic program also supports intracellular replication of Mt in macrophages.

Another detailed metabolic analysis was performed with Mt-infected THP-1 cells (Mehrotra et al., 2014). THP-1 is a human monocytic leukemic cell line which—although defective in p53 (Sugimoto et al., 1992)—is more dependent on FAO and OXPHOS than on glucose and glycolysis for ATP production (Suganuma et al., 2010). This study, albeit performed with a cell line, nevertheless provides some interesting conclusions concerning metabolic reprogramming of host cells in support of Mt replication: (i) The original metabolic program of un-infected THP-1 cells apparently provides suitable conditions for the initial intracellular replication of Mt; this program is clearly different from the TLR-2-mediated metabolic program described above. (ii) However, further metabolic changes occur in the Mt-infected THP-1 cells at later time points. They include enhanced glucose uptake, increased acetyl-CoA synthesis, export of Cit into the cytosol leading to increased FAS and CL biosynthesis. (iii) Suppression of apoptosis observed with host cells infected with virulent, but not with avirulent Mt strains extends the viability of the infected host cells. This is probably linked to the increased glucose uptake which is again observed by the virulent mycobacterial strains only. As intracellular survival and persistence of Mt depend on host-derived FA and CL as nutrients (Pandey and Sassetti, 2008; Lee et al., 2013), the observed induced FA and CL biosynthesis is probably an important prerequisite for the efficient intracellular Mt replication and the establishment of Mt infection. Finally, similar to *Salmonella*, up-regulation rather than down-regulation of p53 seems to occur in Mt*-*infected MPs which may be important for suppression of apoptosis (Galietti et al., 2001; Cruz et al., 2015).

A Warburg-like metabolic shift has also been reported in THP-1 cells infected with *B. abortus* accompanied with an attenuated TCA, reduced amino acid consumption, disrupted mitochondrial functions, and increased lactate production. The pathogen apparently exploits this change in host metabolism to support its own growth and survival (Czyz et al., 2017) in the *Brucella*-containing vacuole (Celli, 2015). Interestingly, the glycolytic enzyme alpha-enolase (ENO-1) interacts with the *Brucella* type IV effector protein BPE123 and this interaction appears to be an important prerequisite for *B. abortus* intracellular replication in HeLa cells (Marchesini et al., 2016). Whether there is a link between this interaction and the observed shift to a Warburg-like metabolism in the *Brucella*-infected THP-1 cells is difficult to decide as in both studies cell lines of different origin are used as host cells. While HeLa cells, like most cancer cell lines, perform already aerobic glycolysis and reduced OXPHOS due to inactive p53 (Berglind et al., 2008), THP-1 depends more on FAO and OXPHOS (Suganuma et al., 2010). The observed Warburg effect in the *Brucella*-infected THP-1 cells therefore suggests a *Brucella* induced shift from OXPHOS to glycolysis (possibly by a mechanism similar to that observed in *Legionella*-infected primary MPs—see above), whereas the ENO-1/BPE123 interaction in HeLa cells may be necessary for the maintenance of the induced glycolytic pathway in the *Brucella*-infected cells. Thus, both observations suggest an essential role of aerobic glycolysis for providing glucose as essential nutrient for intracellular *Brucella* replication. This assumption is in line with a report showing that *B. abortus* survives and replicates in alternatively activated MPs (AAMs). In these host cells, *Brucella* activates PPARγ which leads to increased intracellular glucose and promotes bacterial replication and survival. Glucose uptake by intracellular Brucellae appears to be crucial for their replication in AAMs, as inactivation of the bacterial GLUT, GluP, inhibits intracellular survival in these host cells (Xavier et al., 2013).

Autophagy also seems to support *Brucella melitensis* survival in murine MPs (RAW264.7) (Guo et al., 2012) similar to *L. pneumophila* (Castrejón-Jiménez et al., 2015), possibly by providing additional nutrients (Steele et al., 2015). However, the canonical Atg5-dependent macroautophagic pathway is dispensable for replication of *B. melitensis* and *B. abortus* in fibroblasts (Hamer et al., 2014).

The obligate intracellular pathogen *C. burnetii*, the causative agent of human Q fever, targets *in vivo* primarily alveolar MPs and replicates within these host cells in a lysosome-like parasitophorous vacuole. Little is known on metabolic reprogramming of these primary host cells by *C. burnetii* infection. However, using THP-1 cells it has been shown that sustained activation of Akt is required for intracellular replication of this pathogen and maintenance of host cell viability (Voth and Heinzen, 2009; Hussain and Voth, 2012). Autophagy also seems to support intracellular replication of *C. burnetii* (Castrejón-Jiménez et al., 2015).

Substantial work has been carried out to analyze host cell responses upon infection by *C. trachomatis* and *C. pneumoniae*. These studies demonstrate activation of the PI3K/Akt pathway (Coombes and Mahony, 2002; Sarkar et al., 2015; Subbarayal et al., 2015), subsequent down-regulation of the tumor suppressor p53 (Gonzalez et al., 2014; Siegl et al., 2014), stabilization of HIF-1α (Rupp et al., 2007; Sharma et al., 2011), and activation of Myc (Al-Zeer et al., 2017). Although most of these studies primarily concentrate on strategies that prevent host cell death upon infection, an important prerequisite for intracellular chlamydial proliferation (Byrne and Ojcius, 2004), some of these studies recognize the link between the anti-apoptotic effect and the generation of metabolic programs in the infected host cells that support efficient intracellular chlamydial replication.

*Chlamydia trachomatis* activates the PI3K/Akt pathway which increases the phosphorylation of the E3 ubiquitin-protein ligase leading to proteasomal degradation of p53 (Gonzalez et al., 2014). Down-regulation of p53 in *C. trachomatis* infected HeLa cells enhances PPP which seems to be important for intracellular chlamydial growth (Siegl et al., 2014). *Chlamydia*-infected fallopian tube organoids show Myc activation which induces host cell HK-2, its translocation to the mitochondria and a Warburg-like effect (Al-Zeer et al., 2017). Recently, a whole genome RNA interference screen combined with metabolic profiling identified numerous metabolic targets (Rother et al., 2018). Elevated levels of pyruvate, lactate, and Glu indicated again a shift toward a Warburg-like metabolism. Pyruvate dehydrogenase kinase 2 (PDK2), a key enzyme regulating aerobic glycolysis, was identified as essential host factor for chlamydial replication and development (Rother et al., 2018). Intriguingly, these results were obtained in HeLa cells which already carry out a Warburg-like metabolism, indicating that aerobic glycolysis and a hypermetabolic state is supportive for *Chlamydia* replication.

*Bartonella henselae*, the etiologic agents of bacillary angiomatosis (BA), activates HIF-1 in HeLa cells and in BA-induced tissue lesions *in vivo*. The HIF-1 activation triggers a proangiogenic host cell response with increased levels of vascular endothelial growth factor (VEGF) and up-regulation of genes for the GLUT-3 and glycolytic enzymes (Kempf et al., 2005) thereby inhibiting apoptosis. These conditions probably also favor the intracellular replication of *B. henselae*.

Lastly, it should be noted that type I and type II IFNs are induced by infections of all IBPs described above (Kearney et al., 2013; Snyder et al., 2017). However, while in viral infections IFNs in general trigger antiviral responses (see above), their role in the IBP infections appears to be more complex and enigmatic, i.e., anti-bacterial. On the other hand, also host-detrimental and pro-bacterial effects, especially by IFN-I, have been observed depending on the IBP, the host and the route of infection (Perry et al., 2005; Kearney et al., 2013; Dussurget et al., 2014; McNab et al., 2015; Boxx and Cheng, 2016; Kovarik et al., 2016; Snyder et al., 2017). IFN-mediated events directly affecting the host cell and/or the intracellular IBP metabolism and thereby leading to pro- or anti-microbial effects have hardly been reported and will not be discussed here in detail (Mellor and Munn, 2004; Burke et al., 2014; Chacko et al., 2014; Maggio et al., 2015; York et al., 2015; Fritsch and Weichhart, 2016; Travar et al., 2016; Wu et al., 2016; Saas et al., 2017; Snyder et al., 2017).

In summary, the activation of possible metabolic regulators, including several oncogenes (e.g., PI3K, Akt, Myc, HIF-1), and the inactivation of tumor suppressors (especially p53) within infected host cells have been shown for several IBPs (Figure 6 and Table 3). The link of these processes to immune responses, autophagy and cell death has been rather extensively analyzed. These events are expected to also lead to metabolic reprogramming of the infected host cells which may support the intracellular IBP replication. However, up to now rather little information exists regarding this important aspect. IFN (in particular IFN-I)-induced metabolic processes may positively and negatively influence the intracellular replication of IBPs, but again, so far little is known on these IFN-mediated metabolic processes and their impact on IBP infections.

## Does Metabolic Reprogramming of Host Cells Contribute to Virus/Bacteria Co-Infections?

Virus/bacteria co-infections are frequently observed events causing severe health problems (see e.g., Griffiths et al., 2011; Hendaus et al., 2015). IBPs are common bacterial partners in such co-infections. Frequent virus/IBP co-infections include Mt with HIV (Pawlowski et al., 2012; Bell and Noursadeghi, 2018), *C. trachomatis* with herpes virus (Vanover et al., 2008), and *Staphylococcus aureus* and *L. pneumophila* with influenza virus (Rizzo et al., 2010; Joseph et al., 2013). It is generally agreed that multifactorial processes involving interactions of yet poorly defined host, viral and bacterial factors are responsible for the emergence of these co-infections (McCullers, 2011, 2014; Jamieson et al., 2013). However, little attention has been paid to the possibility that metabolic reprogramming in host cells triggered by the viral partner might support co-infection by the bacterial partner.

Several possibilities are conceivable how metabolic alterations could promote virus/IBP co-existence within the same host cells when both pathogens infect the same organ or tissue, e.g., (a) The first pathogen reprograms upon infection the cell metabolism shaping the cellular immunity and thereby weakens the immune defense against a co-infecting partner. (b) The metabolic reprogramming of host cells triggered by the first pathogen facilitates the adhesion, uptake and/or replication of a second pathogen. (c) The co-infection transforms an active replicative state of the first partner into a stable persistent state. While (a) is a generally accepted mechanism to explain co-infections (Bell and Noursadeghi, 2018), little experimental evidence exists for (b) and (c).

IAV predominantly infects respiratory epithelial cells. *Streptococcus pneumoniae*, a mainly extracellular pathogen (Belon and Blanc-Potard, 2016), is a frequently observed co-infecting partner. The IAV infection causes release of free host sialic acid which serves as nutrient for the co-infecting *S. pneumoniae* and promotes its replication and spread in the lung (Siegel et al., 2014). In addition, IAV infection leads in these cells to an increase in c-Myc and associated with it to enhanced glycolysis and glutaminolysis (Smallwood et al., 2017). This reprogrammed cell metabolism most likely leads to enhanced production and release of lactate which is also a valuable energy and carbon source for *S. pneumoniae*.

The reprogramming of a low-active OXPHOS metabolic state into an aerobic glycolytic state with high glucose consumption and lactate production is a frequent event that occurs in primary host cells upon viral (especially oncoviral) infection (Noch and Khalili, 2012; Mushtaq et al., 2016). This metabolic state is similar to the highly active metabolism observed in many established (often virus-transformed) cell lines which (as pointed out above) are in general excellent host cells for most IBPs. Furthermore, lactate may serve as an efficient energy source for some IBPs when taken up and converted to pyruvate (see above). It is therefore not unlikely that *in vivo* viral infections prepare a lusciously filled dining table for special bacterial guests which reach the virus-infected cells. So far there are, however, only hints and less hard facts supporting this hypothesis (Seganti et al., 1994; Deka et al., 2006, 2007; Prusty et al., 2012; McCullers, 2014; Sixt and Kroemer, 2017).

## Conclusions, Problems and Perspectives

Intracellular pathogens in general must reprogram the metabolism of their host cells to proliferate or persist in these infection niches. This is achieved by viruses mainly by the interaction of diverse, in part well-defined viral factors with specific metabolic control elements of the host cell, especially oncoproteins (oncogenes) and tumor suppressors, or by the introduction of viral oncogenes into the host cell as in case of oncogenic viruses. The metabolic reprogramming often leads in the infected host cells to enhanced glucose uptake, aerobic glycolysis, production and secretion of lactate together with reduced activity of the TCA and OXPHOS (i.e., the characteristic “Warburg effect”). In some cases, Gln may serve as additional or predominant carbon substrate which replenishes the TCA through glutaminolysis. Enhanced FAO combined with OXPHOS represents a less frequently used catabolic alternative. The induced catabolism allows activation of anabolic pathways necessary for the production of the viral nucleic acids, capsids, and eventually membrane envelopes.

Compared to the already advanced knowledge concerning the metabolic reprogramming of virus-infected cells, the corresponding cellular processes that are triggered by IBPs to allow their efficient replication are still poorly understood. The reasons for this problem are diverse: In contrast to viruses, IBPs carry out an own metabolism that is in a pathogen-specific manner adapted to that of the host cell. The two metabolic fluxes are difficult to separate and to measure simultaneously. Only recently, methods have been developed to monitor IBP and host metabolism in parallel. The most sensitive method is dual RNA sequencing (Westermann et al., 2012, 2017) which allows the precise simultaneous determination of the transcripts of all metabolic genes in both partners by use of relatively few infected cells. However, metabolic fluxes are determined by the respective enzyme activities that are controlled in particular by post-transcriptional and post-translational processes. This means that even precise transcript data are of limited value for the determination of the actual metabolism of the two partners. Metabolomics (Nicholson and Lindon, 2008; Misra et al., 2017; Misra, 2018) is a powerful alternative (or additional) method; but with regard to metabolism its weak points are the precise assignment of crucial metabolites to the two partners and the different stability of the metabolites, which together makes the reconstruction of the precise metabolic fluxes in the two partners again difficult. ^13^C-Isotopolog profiling (Eisenreich et al., 2010; Buescher et al., 2015) using different stable metabolites isolated from the two separated partners allows the rather precise reconstruction of their metabolic fluxes during infection. For example, the MS or NMR analysis of the ^13^C-profiles in bacteria-specific biosynthetic products such as mDAP and (host)-essential amino acids provides unequivocal information about the metabolic fluxes in the bacteria. On the other hand, this method in its present form is not very sensitive (i.e., it requires rather large amounts of biological material, i.e., >10^8^ bacterial cells) and is therefore hardly applicable to *in vivo* studies. This means that the presently available methods only give us a rather rough idea about the metabolic reprogramming of the host cell during IBP infections and about the adapted intracellular metabolism of the IBP.

The second problem concerns the used host cells. Especially for infections caused by IBPs it is often even unclear what the primary host cell and its metabolic state is. Established cell lines, often used as host cell models for IBP and virus infections, have significant drawbacks for investigating metabolic issues related to infections, in particular when analyzing the metabolic reprogramming triggered by the pathogen.

The third and probably biggest problem is the investigation of the metabolism of infected host cells under the changing environmental *in vivo* conditions certainly different from the rich media conditions which are normally applied in *in vitro* studies. Only few of the studies discussed in this review addressed this problem.

These drawbacks already indicate the necessary future developments: Of great importance is clearly the further development of the three mentioned methods, but also the finding of novel techniques which will allow the analysis of the actual cell metabolism of host cell and pathogen under *in vivo* infection conditions. To achieve this goal, it is equally important to introduce genuine primary host cells, tissue and organoid systems as well as suitable animal models for the analysis of the crucial metabolic processes that occur in the host cell and the pathogen during infections. Only armed with this knowledge, we can expect to find metabolic targets for novel drugs that are of therapeutic value in the fight against diseases caused by microbial pathogens.

## Addendum

While this article was in the final reviewing phase, a review was published by A. Best and Y. Abu Kwaik (*Trends Microbiol* Jan 14. pii: S0966-842X(18)30288-9. doi: 10.1016/j.tim.2018.12.012. [Epub ahead of print]) which adds some interesting aspects to the bipartite metabolism of intracellular replicating bacterial pathogens.

## Author Contributions

WG and WE designed the study. WG, WE, TR, and JH wrote the article.

### Conflict of Interest Statement

The authors declare that the research was conducted in the absence of any commercial or financial relationships that could be construed as a potential conflict of interest.

## Abbreviations

AAM: alternatively activated macrophage
ACL: ATP-dependent citrate lyase
ADV: adenovirus
α-KG: α-ketoglutarate
AMPK: AMP-activated protein kinase
BA: bacillary angiomatosis
BMDM: bone marrow-derived MP
CaMKK: Ca^2+^/calmodulin-dependent protein kinase kinase
cDC: conventional dendritic cell
CH25H: cholesterol-25 hydroxylase
ChREBP: carbohydrate-responsive element-binding protein
Cit: citrate
CL: cholesterol
DC: dentritic cell
DENV: dengue virus
EBV: Epstein-Barr virus
ENO: phosphopyruvate hydratase (enolase)
ETC: electron transport chain
FA: fatty acid
FAO: fatty acid oxidation
FAS: fatty acid synthesis
FASN: fatty acid synthase
G6P: glucose-6-phosphate
GAPDH: glyceraldehyde-3-phosphate dehydrogenase
GLS: glutaminase
GLUT: glucose transporter
HCMV: human cytomegalovirus
HCV: hepatitis C virus
HIF-1: hypoxia-inducible transcription factor 1
HIV: human immuno deficiency virus
HK: hexokinase
HSV: herpes simplex virus
HUVEC: human umbilical vein endothelial cells
IAV: influenza A virus
IBP: intracellular bacterial pathogen
IDO: indoleamine-2,3-dioxygenase 1
IFN: interferon
IL: interleukin
ISG: interferon stimulated genes
KS: Kaposi's sarcoma
KSHV: Kaposi's sarcoma-associated virus
Kyn: kynurenine
LCMV: lymphocytic choriomeningitis virus
LCV: *Legionella*-containing vacuole
LD: lipid droplets
LPS: lipopolysaccharide
LpSpl: *Legionella pneumophila* sphingosine-1-phosphate lyase
MCMV: murine cytomegalovirus
mDAP: meso-diaminopimelate
MEF: mouse embryonic fibroblast
MHC: major histocompatibility complex
MO: monocyte
MP: macrophage
Mt: *Mycobacterium tuberculosis*
mTOR: target of rapamycin
mTORC1: target of rapamycin complex 1
MV: measles virus
Myc: myelocytomatosis oncogene
NFAT: nuclear factor of activated T-cells
NF-κB: nuclear factor 'kappa-light-chain-enhancer' of activated B-cells
OAA: oxaloacetate
OXPHOS: oxidative phosphorylation
PBMC: peripheral blood mononuclear cells
PCV: pathogen-containing vacuole
pDC: plasmacytoid dendritic cell
PDK: pyruvate dehydrogenase kinase
PDPK1: 3-phosphoinositide dependent protein kinase-1
PG: peptidoglycan
PI3K: phosphoinositide-3-kinase
PKM2: pyruvate kinase M2
polyI:C: polyinosinic-polycytidylic acid
PPAR: peroxisome proliferator-activated receptor
PPP: pentose phosphate pathway
PTEN: phosphatase and tensin homolog
Pyc: pyruvate carboxylase
RB: retinoblastoma protein
SCV: *Salmonella*-containing vacuole
SGLT1: sodium-glucose cotransporter 1
SIRT: sirtuin
SREBP: sterol regulatory element-binding protein
STAT: signal transducer and activator of transcription
STING: stimulator of interferon genes
Suc: succinate
SV40: simian virus 40
TCA: tricarboxylic acid cycle
TLR: Toll-like receptor
TSC: tuberous sclerosis complex
Vpr: viral protein
VSV: vesicular stomatitis virus.
